# Codon-specific ribosome stalling reshapes translational dynamics during branched-chain amino acid starvation

**DOI:** 10.1186/s13059-025-03800-6

**Published:** 2025-09-27

**Authors:** Lina Worpenberg, Cédric Gobet, Felix Naef

**Affiliations:** Institute of Bioengineering, School of Life Sciences, Ecole Polytechnique Fédérale de Lausanne (EPFL), Lausanne, CH-1015 Switzerland

**Keywords:** Translation regulation, Ribosome profiling, Amino acid starvation, tRNA charging, Codon-specific stalling, Elongation bottleneck, Nutrient stress response

## Abstract

**Background:**

Cells regulate protein synthesis in response to fluctuating nutrient availability through mechanisms that affect both translation initiation and elongation. Branched-chain amino acids, leucine, isoleucine, and valine, are essential nutrients. However, how their depletion affects translation remains largely unclear. Here, we investigate the immediate effects of single, double, and triple branched-chain amino acid deprivation on translational dynamics in NIH3T3 cells using RNA-seq and ribosome profiling.

**Results:**

All starvation conditions increased ribosome dwell times, with pronounced stalling at all valine codons during valine and triple starvation, whereas leucine and isoleucine starvation produced milder, codon-specific effects. Notably, stalling under isoleucine deprivation largely decreased under triple starvation. Positional enrichment of valine codons near the 5′ end and downstream isoleucine codons potentially contributes to these patterns, suggesting a possible elongation bottleneck that influences translational responses under branched-chain amino acid starvation. The presence of multiple valine stalling sites was associated with decreased protein levels. Finally, codon-specific dwell time changes correlated strongly with patterns of tRNA isoacceptor charging.

**Conclusions:**

Together, these findings suggest that differential ribosome stalling under branched-chain amino acid starvation reflects a balance between amino acid supply, tRNA charging dynamics, codon position, and stress-response signaling.

**Supplementary Information:**

The online version contains supplementary material available at 10.1186/s13059-025-03800-6.

## Background

Branched-chain amino acids (BCAAs), namely leucine, isoleucine, and valine, are essential nutrients involved in diverse cellular and physiological processes, including protein synthesis, metabolism, cellular signaling, brain function, and immune responses [1–5]. Because mammalian cells cannot synthesize these amino acids, they rely on external sources to maintain proteostasis and meet metabolic demands. Accordingly, fluctuations in BCAA levels can have strong implications for health and disease [6–8]. Elevated BCAA concentrations are associated with obesity, insulin resistance, and type 2 diabetes mellitus [9–12], while low BCAA plasma levels are correlated with the prevalence of liver cirrhosis and hepatic encephalopathy [13], and shifts in BCAA metabolism have been linked to tumor growth, progression, and survival [14–17].

Translation is one of the most energy- and resource-consuming processes within dividing cells, and thus, is tightly regulated in response to nutrient availability [18]. Two key pathways govern this response: The mechanistic target of rapamycin complex 1 (mTORC1) pathway and the Integrated Stress Response (ISR). The modulation of both pathways aims to reduce global translation initiation to maintain cellular homeostasis and to help cells cope with stress. Under normal conditions, mTORC1 promotes translation initiation by phosphorylating substrates such as eukaryotic translation initiation factor 4E-binding protein 1 (4E-BP1) and ribosomal protein S6 kinase (S6K) [19–21], thereby promoting the translation of transcripts which contain a terminal oligopyrimidine tract (TOP) that encode ribosomal and other translational machinery components [22, 23]. When amino acid levels are limited, mTORC1 activity drops, causing reduced global translation initiation, downregulated translation of TOP transcripts, and partial reprogramming of gene expression. Meanwhile, the General Control Nonderepressible 2 (GCN2) kinase, a central component of the ISR, responds to increased levels of uncharged tRNAs that accumulate when particular amino acids are deficient [24, 25]. This triggers phosphorylation of eukaryotic translation initiation factor 2 subunit 1 (eIF2α), which suppresses cap-dependent initiation while selectively promoting translation of stress-response genes [26–28].

Although many studies have focused on how these pathways downregulate translation initiation, it is increasingly evident that amino acid availability also controls translation elongation. A steady supply of aminoacyl-tRNAs is required for continuous elongation, and a shortage of even one amino acid can trigger codon-specific ribosome stalling [29]. A recent example shows that leucine starvation leads to reduced charging of tRNA^Leu^_UAA_, which causes ribosome stalling at UUA codons and promotes mRNA frameshifting and protein misfolding [30]. This selective charging of tRNA isoacceptors adds another layer of regulation, whereby some synonymous codons become more prone to stalling than others [31, 32]. Besides the availability of charged tRNAs, the sequence context of mRNA itself plays a crucial role in modulating ribosome dynamics during translation as both codon composition and secondary mRNA structures influence translation elongation. Stable secondary structures, such as stem-loops or pseudoknots, can create physical barriers to ribosome progression leading to transient pausing or stalling [33]. Likewise, clusters of rare codons can slow translation by limiting the availability of cognate tRNAs, further affecting elongation kinetics [34]. These pauses not only influence translation efficiency but also impact co-translational protein folding and may serve as regulatory checkpoints, particularly under conditions of nutrient limitation [35–38]. This phenomenon underscores the complexity of codon-level control under nutrient stress.

Technical advances, including ribosome profiling (Ribo-seq), have made it possible to study translation at single-codon resolution [39]. By isolating and sequencing ribosome-protected mRNA fragments (RPFs), one can capture a snapshot of ribosome occupancy across the transcriptome, wherein the density of reads at a given codon is inversely proportional to the local speed of translation. Thus, this allows us to detect where ribosomes pause during normal growth or under stress. In particular, codon-specific ribosome dwell times (DT) can be inferred from Ribo-seq data, serving as an indicator of how long the ribosome remains at each codon [40].

While previous research has highlighted the impact of individual amino acid shortages on the translation dynamics and that the effects vary depending on the specific amino acid being limited [41–43], a comprehensive understanding of how simultaneous BCAA limitation influences translation is lacking. Here, we address this gap by systematically employing Ribo-seq upon single and simultaneous starvation of BCAAs to assess global translational output and codon-specific dwell times. This approach allows us to identify unique stalling patterns associated with each BCAA deprivation. Complementary quantitative proteomics experiments allowed us to correlate these translational modifications with changes in protein abundance. Additionally, tRNA charging assays elucidated the mechanistic relationship between amino acid depletion, uncharged tRNA isoacceptors, and ribosomal pausing. Through the integration of these multi-omics datasets, we demonstrated that BCAA starvation induces diverse translational control patterns, reflecting the interplay of mTORC1 and GCN2 pathways, tRNA isoacceptor availability, and transcript codon usage. Additionally, our analysis highlights a positional effect within transcripts, indicating that translational regulation is not only influenced by the codon composition of transcripts but also by the location of codons within the mRNA sequence. This study uncovers a previously underappreciated layer of complexity in BCAA-specific translational regulation, setting the stage for future investigations into the precise mechanisms that emerge when valine, isoleucine, and leucine are concurrently limited.

## Results

### Translation elongation rates are modulated in a codon-specific, non-additive manner upon limitation for BCAAs

To systematically assess how BCAA limitation affects translation elongation, we performed Ribo-seq on NIH3T3 cells starved for BCAAs, namely leucine, isoleucine, and valine. We examined the effects of single amino acid starvations (-Leu, -Ile, and -Val), as well as combinations, including a double starvation of leucine and isoleucine (hereafter referred to as “double”) and a starvation of leucine, isoleucine, and valine (“triple”), allowing us to identify potential synergistic effects. Our experiments focused on a 6-h time window to capture early cellular responses and avoid long-term adaptations or cell death. Across these Ribo-seq experiments, footprint lengths peaked at 31 nucleotides and reads predominantly mapped to coding sequences (Additional file 1: Fig. S1A-B). To examine how the limitation of BCAAs alters elongation, we inferred the genome-wide DT changes for all 61 sense codons across the different starvation conditions using our Ribo-DT pipeline (Additional file 2: Table S1) [49]. Under control conditions, the DTs in our NIH3T3 cells were correlated to those previously reported in mouse liver, indicating that our system robustly captures mammalian translation elongation and is suitable for controlled nutrient-deprivation studies [40] (Additional file 1: Fig. S1C). Moreover, we noticed that DT changes extend beyond the ribosomal A-site, including the P-site, E-site, and even further positions (Additional file 1: Fig. S2A), consistent with other studies on single amino acid starvation [43] (Additional file 1: Fig. S2B-C). Consequently, we focused on a ± 3 codon region centered on the P-site rather than a single codon position.

**Fig. 1:**
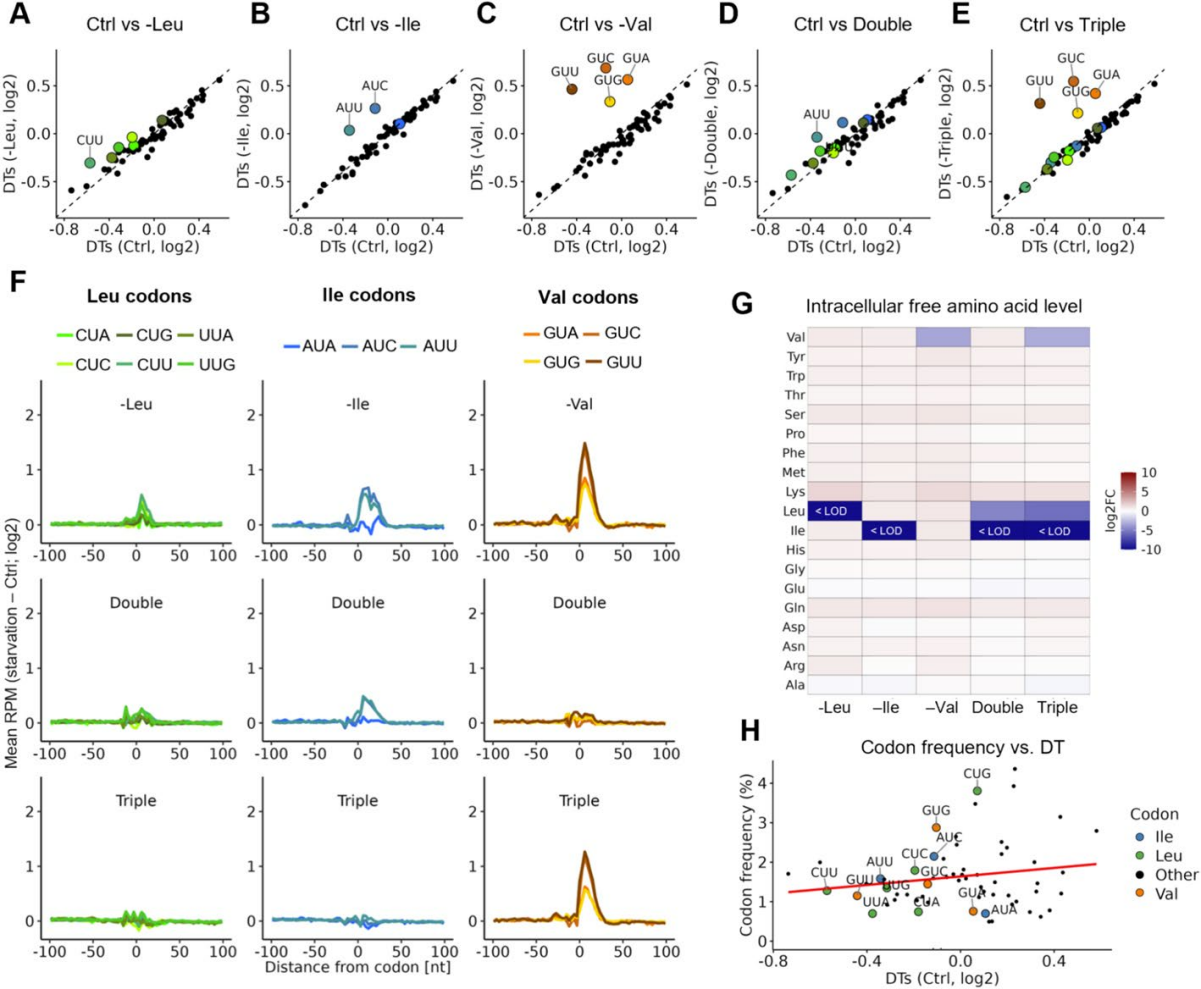
Translation elongation rates are modulated in a codon-specific, non-additive manner upon limitation for BCAAs. **A–E** Codon-specific ribosomal dwell times (DTs) in Ctrl vs. starvation (Double: -Leu & -Ile, Triple: -Leu & -Ile & -Val). Shown is the mean DT of triplicates over a ± 3 codon region centered on the P-site. Significance was evaluated with ANOVA followed by Tukey’s HSD test. Annotated are codons with significantly upregulated DT relative to Ctrl (*p*-value < 0.01). **F** Changes in Ribo-seq read density around annotated codons relative to the Ctrl. Mean reads per million (RPM) values were normalized to Ctrl condition, and the mean log_2_FC computed for a ± 100 nt window around codons. **G** Heatmap of log_2_FC in intracellular amino acid levels under different starvation conditions relative to Ctrl. Values below detection limit (LOD) are indicated. **H** Scatter plot of DT under Ctrl condition (**A–E**, log₂) vs. codon frequency (%). Codons for Val (orange), Ile (blue), and Leu (green) are highlighted. A linear trendline (red line) shows the relationship between DT and codon frequency

Intriguingly, only two of the three isoleucine codons (AUU and AUC) showed increased DTs upon Ile starvation (*p* < 0.01), while just one leucine codon (CUU) exhibited a modest but significant DT increase (*p* < 0.01) under Leu starvation (Fig. 1A,B, Additional file 1: Fig. S2A). In contrast, all four valine codons exhibited significantly increased DTs upon Val starvation (*p* < 0.01; Fig. 1C). The DT changes were milder under double starvation, with only AUU (Ile) showing a significant DT increase among the BCAA codons (*p* < 0.01; Fig. 1D). Surprisingly, under triple starvation, only valine codons exhibited significant DT increases (*p* < 0.01), with no significant changes observed for any of the leucine or isoleucine codons (Fig. 1E). We complemented these observations by examining the averaged Ribo-seq read density around each codon corresponding to the starved amino acid. Under Val starvation, ribosomes strongly accumulated around all valine codons, whereas Leu starvation produced only minor peaks near CUU, CUC, and UUG, which diminished further during double or triple starvation (Fig. 1F). Ile starvation caused a strong enrichment of ribosome density around AUC and AUU codons (Fig. 1F). Notably, these enrichments persisted under double starvation but disappeared under triple starvation. These starvation-specific DT and ribosome density modulations were also evident at the individual transcript level, as exemplified by *Col1a1*, *Col1a2*, *Aars*, and *Mki67*, which showed persistent Val-codon-specific ribosome density increases but lost Ile-codon-specific increases under triple starvation (Additional file 1: Fig. S3A-D & 4A-D).

The observation that increased DTs were seen exclusively on valine codons under triple amino acid starvation was highly unexpected. To gain further insight into why valine codons uniquely displayed increased DTs under triple starvation and to exclude the possibility of compensatory mechanisms, we measured free intracellular amino acid levels (Additional file 3: Table S2). The deprived amino acids were drastically reduced or even undetected in their respective starvation conditions (Fig. 1G; Additional file 1: Fig. S5A), confirming effective cellular starvation. While valine appeared slightly less depleted than leucine or isoleucine, this is likely due to technical variation in detection sensitivity. We next investigated whether differential codon usage frequency explained the observed DT changes. However, codons showing prolonged DTs were not particularly rare in transcripts expressed in mouse NIH3T3 cells (Additional file 1: Fig. S5B). Moreover, there was neither a general correlation between DTs or DT changes in comparison to Ctrl (∆DTs) and average codon frequency nor a bias in the effects of starvation on the DTs toward codons considered “slow” or “fast” (Fig. 1H, Additional file 1: Fig. S5C-G). Thus, the codon-specific stalling under BCAA starvation appears not to be driven by overall codon usage.

### Stress response pathways are differentially activated upon leucine, isoleucine, and valine starvation

Prior work suggests that the activation of stress response pathways, particularly the GCN2 and mTORC1 pathways, can induce ribosome stalling during amino acid starvation [43]. In particular, a strong activation of these stress pathways (e.g., under leucine deprivation) prevented ribosome stalling by efficiently reducing global translation load, whereas weaker modulations (e.g., arginine deprivation) led to more frequent stalling. Therefore, we evaluated the activation of stress pathways across our BCAA starvations.

RNA-seq and Ribo-seq revealed broad activation of stress response pathways across all starvation conditions. PCA of mRNA levels, ribosome footprints (RPFs), and ribosome densities (RD = Ribo-seq/RNA-seq) showed shared transcriptional and translational reprogramming (Additional file 1: Fig. S6A-B; PC1), particularly involving the UPR, ubiquitin-like protein binding, and cell cycle regulation (sister chromatid segregation) (Additional file 1: Fig. S6D-E, Additional file 4: Table S3), all core elements of the cellular stress response. Val and triple starvation clustered closely and furthest from control, especially at the RPF level, indicating stronger translational effects (Additional file 1: Fig. S6B). Ile starvation displayed a distinct pattern, most evident in Ribo-seq and even more in RD space (Additional file 1: Fig. S6B-C), separating from control only along PC2. The transcripts driving this separation were enriched for stress-responsive transcription and cytoplasmic translation genes (Additional file 1: Fig. S6F, Additional file 4: Table S3). Pairwise comparisons confirmed that Val starvation caused the most pronounced RD shifts: 423 transcripts increased and 403 decreased (Additional file 1: Fig. S7A, Additional file 4: Table S3), largely overlapping with triple starvation. Ile starvation, by contrast, affected a unique subset, notably cytoplasmic ribosomal protein genes (Additional file 1: Fig. S7B, D). Transcripts with altered RD under all BCAA starvations included translation initiation factors (*Eif4g3*, *Eif1*), transcription factors, and UPR-associated genes (*Chop*, *Atf4*, *Ppp1r15a*, *Yod1*) (Additional file 1: Fig. S7B-C).

**Fig. 2:**
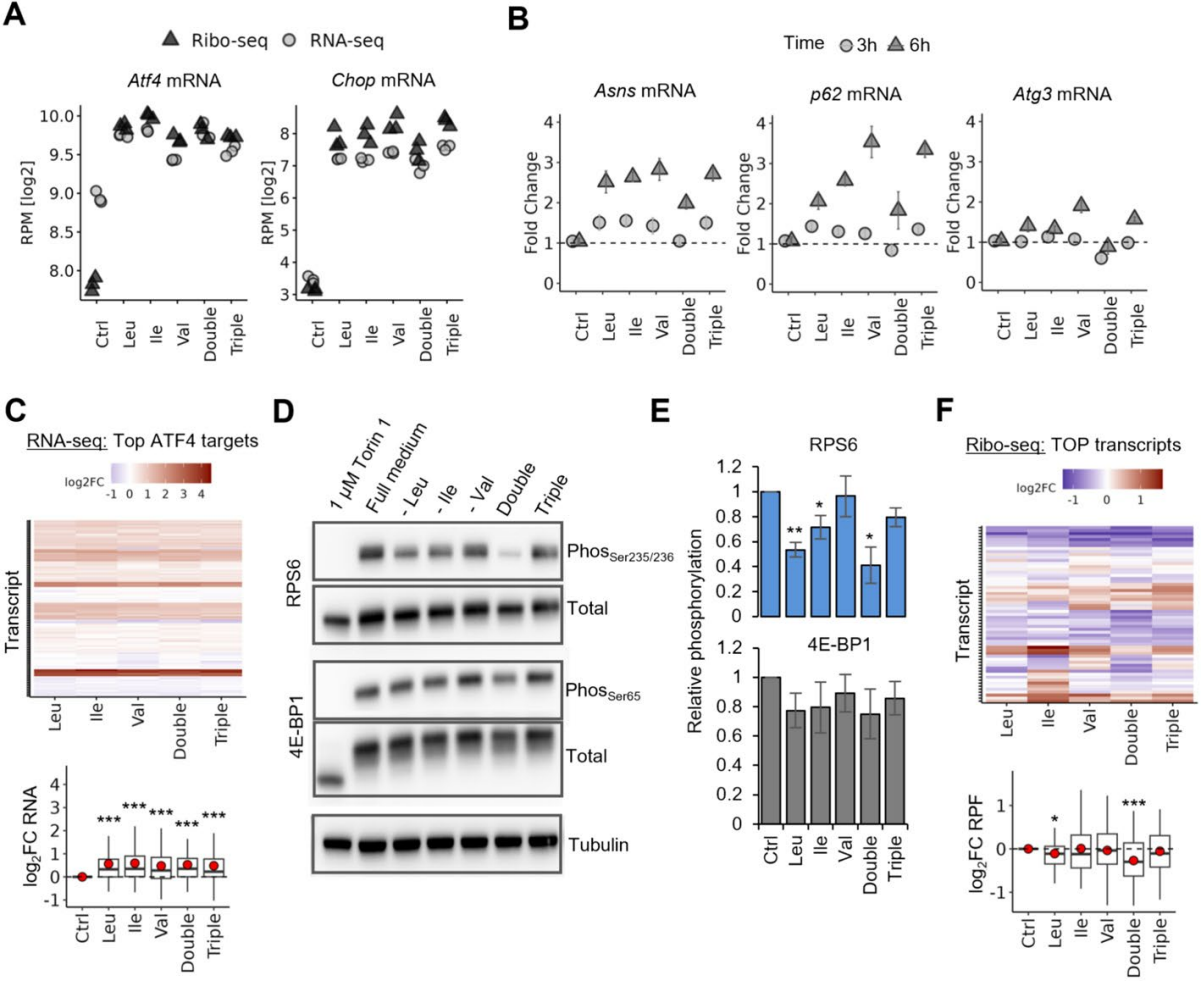
Stress response pathways are differentially activated upon Leu, Ile, and Val starvation. **A** Expression levels of *Atf4* and *Chop* in RNA-seq and Ribo-seq under different deprivation conditions. Values represent log₂-transformed reads per million (RPM). Each point represents an individual replicate. Statistical significance was determined by unpaired *t*-tests comparing each starvation to Ctrl, with all comparisons resulting in *p* < 0.01. **B** qPCR analysis of mRNA levels for *Asns*, *p62*, and *Atg3* under different starvations at 3 h and 6 h. Expression levels were normalized using the ΔΔCt method (normalized to *Gapdh* expression) and are shown as fold change relative to Ctrl. Bars represent mean fold change, with error bars indicating standard error of mean (SEM). **C** Heatmap and quantification of log₂FC for the top 150 ATF4 target genes from ChIP-Atlas [55] at the mRNA level. Genes are clustered based on their expression profiles using *k*-means clustering (*k* = 12). In the boxplot, red points indicate the mean expression change. Significance was assessed using unpaired *t*-tests. **D** Representative Western blot showing phosphorylation levels of 4E-BP1 (Phos_Ser65_) and RPS6 (Phos_Ser235/236_) under different amino acid starvations. Total protein levels and Tubulin level serve as loading controls. 1 µM Torin1 treatment and full medium conditions are included as controls. **E** Quantification of phosphorylation levels of 4E-BP1 and RPS6, normalized to total protein levels and relative to the control (Ctrl). Quantification was performed using ImageJ. Data represent mean ± SEM from three independent experiments. Statistical significance was determined using unpaired *t*-tests. **F** Heatmap and quantification of log₂FC for the known TOP motif containing transcripts [22] in our Ribo-seq. Genes are clustered based on their expression profiles using *k*-means clustering (*k* = 12). In the boxplot, red points indicating the mean expression change. Significance was assessed using unpaired *t*-tests

CHOP and ATF4 are stress-induced transcription factors mediating the GCN2 pathway. We observed strong upregulation of *Atf4* and *Chop* at both mRNA and ribosome footprint levels across all starvations (Fig. 2A), indicating robust ISR activation. This was supported by qPCR profiling of ISR genes (*Asns*, *Atg3*, *p62*) over a 3–6 h time course, with two showing strong induction at 6 h under all starvations (Fig. 2B). Notably, double starvation showed the weakest ISR gene activation and lower *Chop* induction (Fig. 2A,B). Still, RNA-seq confirmed significant upregulation of direct ATF4 targets across all conditions without major differences (Fig. 2C). To assess mTORC1 activity, we measured RPS6 and 4E-BP1 phosphorylation by Western blot. Double starvation, and to a lesser extent Leu and Ile starvation, reduced RPS6 phosphorylation, indicating strong mTORC1 inhibition, whereas Val and triple starvation had milder effects (Fig. 2D,E; Additional file 1: Fig. S8). A similar but less pronounced pattern was seen for 4E-BP1 (Fig. 2D,E). Consistently, ribosome coverage of mTORC1-sensitive TOP transcripts was reduced under Leu and double starvation, but not consistently under Val or triple starvation (Fig. 2F). Because TOP transcripts, which mainly encode ribosomal proteins (RPs), often involve multimapped reads, we reanalyzed Ribo-seq data including them (Additional file 1: Fig. S7E). This confirmed the trend of strongest downregulation under double starvation, with milder effects from Leu and Ile starvation. Thus, while all starvations triggered stress responses, the exact balance of GCN2 and mTORC1 pathway engagement differed depending on which BCAAs were depleted. This differential pathway activation may play a role in why the extent of effects observed on the ribosome DTs is milder during double and Leu starvation.

### BCAA starvations differentially modulate translation initiation and elongation

**Fig. 3:**
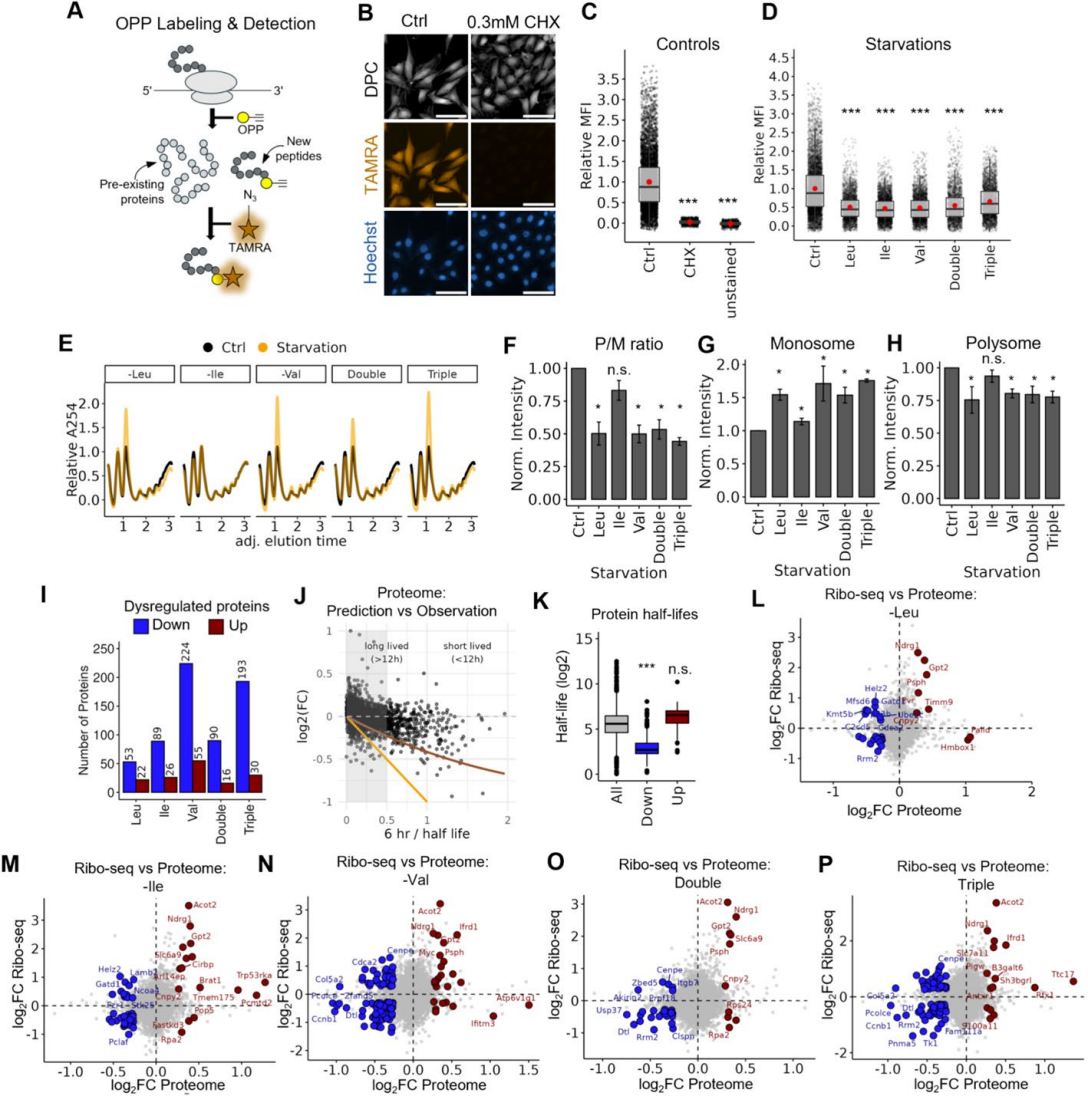
BCAA starvations differentially modulate translation initiation and elongation. **A** Schematic representation of detection of newly synthesized proteins by OPP incorporation assay. Newly synthesized proteins incorporate OPP, which causes translation termination and allows subsequent labeling with TAMRA-azide. This enables their detection and quantification via fluorescence microscopy, distinguishing them from pre-existing proteins that lack OPP. **B** Representative fluorescence microscopy images of TAMRA and Hoechst staining. Wild-type cells and cells treated with 0.3 mM CHX to inhibit translation are shown. TAMRA (orange) represents OPP staining, marking newly synthesized proteins. Hoechst stains nuclei (blue), and DPC (digital phase contrast) provides cellular morphology. Scale bar: 100 µm. **C,D** Quantification of OPP incorporation under **C** Ctrl and **D** starvation conditions. Boxplot representations display the relative mean fluorescence intensity (MFI) of the TAMRA signal; each dot represents an individual cell, and red points indicate the mean MFI. Data represent three biological replicates. Statistical significance between conditions was determined using unpaired *t*-tests with Bonferroni correction. **E** Representative polysome profiles of Ctrl (black line) and starvations (orange line). **F–H** Quantification of polysome-to-monosome (P/M) ratio (**F**), monosome (**G**), and polysome (**H**) fractions. **I** Number of dysregulated proteins (*p* < 0.01 and |log₂FC|> 0.26) across starvations. **J** Correlation between measured log₂FC proteomic changes upon Triple starvation and published protein half-lifes in wildtype NIH3T3 cells [59]. Lines indicate theoretical log_2_FC assuming standard degradation kinetics under total synthesis block (orange) and 50% synthesis block (sienna). **K** Distribution of protein half-lives (see **J**) for down- and upregulated proteins under any BCAA starvation. **L–P** Scatter plots showing the relationship between log_2_FC measured in proteome and Ribo-seq under the given conditions. Red dots represent transcripts corresponding to upregulated proteins (*p* < 0.01 and log₂FC > 0.26) with significant changes in Ribo-seq (*p* < 0.01 and |log₂FC|> 0.26), while blue dots highlight transcripts corresponding to downregulated proteins (*p* < 0.01 and log₂FC < − 0.26) with significant changes in Ribo-seq (*p* < 0.01 and |log₂FC|> 0.26), indicating translational repression

Because both the modulation of the GCN2 and mTORC1 pathways tend to decrease translation initiation, we examined to what extent overall protein synthesis rates were reduced under each starvation. We treated cells with a short pulse of OPP (a puromycin alkyne analog) after the starvation periods, then used click-chemistry to fluorescently tag newly synthesized proteins with TAMRA-azide for quantification [57] (Fig. 3A). Cells treated with cycloheximide (CHX), a translation inhibitor, and OPP-untreated cells confirmed that the observed fluorescence signal was due to active translation (Fig. 3B,C). In all starvation conditions, TAMRA signal intensity was significantly reduced, confirming a decrease in global protein synthesis rates (Leu: 50%, Ile: 46%, Val: 48%, Double: 54%, Triple: 65%; Fig. 3D).

As a parallel approach to analyze global translation, we performed polysome profiling (Fig. 3E). We observed that starving cells for Leu, Val, and their combinations lead to significant increases in monosome levels and a decrease in polysomes, as evidenced by a reduced polysome-to-monosome (P/M) ratio of about 50% (Fig. 3F–H), indicating globally fewer ribosomes per transcript. By contrast, the starvation of Ile alone did not alter the P/M ratio or the levels of polysomes (Fig. 3F, H), despite reduced OPP incorporation (Fig. 3D). This suggests that translation under -Ile starvation may be particularly affected at the elongation step. It is important to note that polysome profiles reflect the combined effects of both initiation and elongation: while reduced initiation decreases the number of ribosomes per mRNA and thus lowers polysome levels, elongation defects can lead to ribosome accumulation on transcripts through ribosome queuing and stalling, resulting in stabilized or elevated polysome levels. However, such accumulation is not a universal outcome and depends on the specific nature of elongation perturbation. For instance, stalling events close to the CDS start or conditions with strongly reduced initiation rates typically prevent ribosome buildup and thus polysome accumulation. In our data, DT analysis (Fig. 1) indicates that elongation modulations also occur under Val and Triple starvation. However, this was not reflected by an increase in polysome levels, suggesting that the nature of elongation impairment differs from that seen under Ile starvation. These findings suggest that BCAA starvation affects both initiation and elongation, but the specific translation patterns vary by condition and are driven by different underlying mechanisms.

### BCAA starvation remodels the proteome

To determine whether the identified changes in global protein synthesis are already reflected at the proteome level, we performed TMT-based quantitative proteomics. Among 7004 proteins identified (6177 also covered in RNA-seq and Ribo-seq; Additional file 4: Table S3), Val and triple starvation caused the largest proteome changes (224/193 downregulated proteins, respectively; Fig. 3I), while Leu starvation had the mildest effect (53 downregulated, 22 upregulated; Fig. 3I). Given our relatively short 6 h starvation period and the generally long half-lives of proteins, only moderate changes in protein abundance were expected as shown by a simple kinetic model. Indeed, we modelled log_2_FC values under a scenario where protein synthesis is reduced, and assuming that protein stability is not strongly affected after 6 h of starvation. Using published half-lives in 3T3 cells [59], we found that the measured protein FCs on average depend on the half-lives as predicted by the model (Fig. 3J,K).

Next, we investigated whether the altered protein output aligned with ribosome occupancy, by comparing Ribo-seq and proteomics data (Fig. 3L–P). Many of the downregulated proteins showed a decreased level of ribosome footprints (Fig. 3L–P; blue dots), and upregulated proteins showed an increase in the footprint level (Fig. 3L–P; red dots), aligning with the hypothesis of global translation initiation modulation. However, a subset of downregulated proteins maintained or even increased the ribosome occupancy of the corresponding transcript (Fig. 3L–P; blue dots), implying that the changes in protein abundance did not correlate with the ribosome footprint data. Given the observed increased DTs (Fig. 1), a plausible explanation for the increased ribosome footprints is ribosome stalling. Such stalling could impair effective protein synthesis despite sustained or even increased ribosome occupancy. Importantly, it is also possible that initiation is suppressed while stalled ribosomes accumulate, leading to stable or elevated RPF signals without productive translation. Increased protein degradation could likewise explain the decreased protein output despite increased ribosome footprints. However, the observed proteomic log_2_FC of downregulated proteins were often milder than expected based on their half-life under a normal condition (Fig. 3J), which would be difficult to reconcile with a dominant contribution from global protein destabilization. Nonetheless, a contribution from protein degradation cannot be excluded. Therefore, our data point to a complex interplay between altered initiation, elongation, and potentially protein turnover: Val starvation is characterized by strong, positionally biased ribosome stalling. Leu starvation appears to primarily impact translation initiation, likely via mTORC1 repression, and Ile starvation shows a mixed phenotype, with features of both impaired initiation and codon-specific elongation delays. While protein stability changes or degradation may still contribute as well to the observed changes in protein output, our current data do not allow for quantitative comparisons of proteolytic effects.

### BCAA starvation leads to ribosome density shifts toward the 5′ of the CDS

**Fig. 4:**
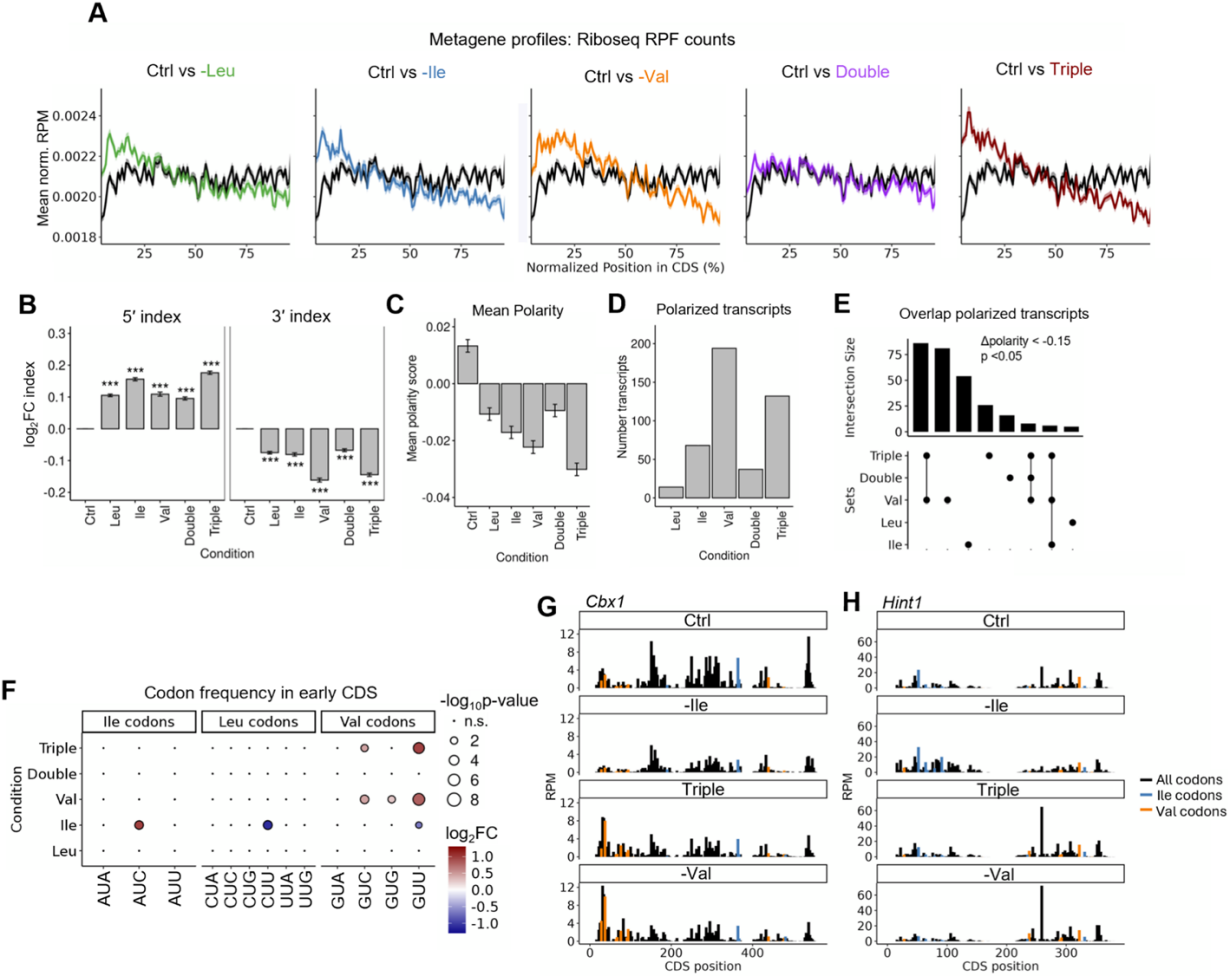
BCAA starvation leads to ribosome density shifts toward the 5′ of the CDS.** A** Metagene profiles showing the mean normalized RPF count of the Ribo-seq along the CDS for each starvation (colored) compared to Ctrl (black). Solid lines represent the mean signal at each normalized CDS position, and shaded ribbons indicate the standard error of the mean (SEM). **B** Bar plot quantifying the regional “ramp” in ribosome densities. Log₂-transformed ratios of summed RPF counts in the 5′ region (1–20% of CDS positions), and 3′ regions (80–100% of CDS positions) are shown for starvation relative to Ctrl. Bars indicate the mean value with SEM as error bars. Statistical significance between Ctrl and starvation was determined using unpaired *t*-tests with Bonferroni correction. **C** Bar plot indicating the mean transcript-specific polarity scores in the indicated conditions. **D** Bar plot indicating the number of transcripts displaying a significant reduction of the polarity score (Δpi < − 0.15; *p* < 0.05) relative to the control condition upon indicated starvations. **E** UpSet plot summarizing the overlap of transcripts with significant 5′ polarity score shifts (Δpi < − 0.15; *p* < 0.05) across BCAA starvations. Each bar in the upper panel represents the number of transcripts showing polarity shifts in the conditions indicated by the connected black dots below. Single dots indicate condition-specific sets, while connected dots indicate shared transcripts between multiple starvations. **F** Dot plot showing the log₂ fold change in mean codon frequencies for transcripts exhibiting a significant 5' polarity shift. For each condition, the codon frequencies within the first 20% of the CDS of polarized transcripts were compared to the frequencies in the same region of all other expressed transcripts. Dot size reflects the − log₁₀(*p*-value) from unpaired *t*-tests (BH-adjusted), and color indicates the magnitude and direction of the log₂ fold change. **G,H** Representative ribosome profiling tracks for the genes *Cbx1* (**G**) and *Hint1* (**H**), with Val and Ile codons annotated

Our Ribo-seq data (Fig. 1) showed increased DTs at all or a subset of codons cognate to the limiting amino acid and our global protein synthesis quantifications (Fig. 3) indicated that BCAA starvation lowered protein output while also impacting elongation. To further investigate the regional distributions of ribosome footprints and potential stalling sites, we next performed metagene positional analysis of all ribosome footprints. Importantly, the first/last 15 nt of each transcript were excluded from the analysis to avoid distinct effects associated with the start/stop codon peaks [58]. Under all BCAA starvations, we observed a 5′ ribosome footprint polarization (Fig. 4A), a pattern that can be observed under various stress conditions and perturbations [60–62]. This general 5′ bias likely reflects a combination of slowed elongation and altered ribosome dynamics and is not necessarily linked to codon-specific stalling. However, by computing differential ribosome occupancies in the first and last 20% of CDSs (5′ and 3′ indices), we found that Val and Triple starvation show a much stronger and more asymmetric polarization, characterized by pronounced 5′ accumulation and 3′ depletion of ribosome density (Fig. 4B). We also calculated polarity scores, capturing ribosome bias along the full CDS, which revealed a modest shift toward the 5’ across all starvations but most under Val and triple conditions (Fig. 4C; Additional file 1: Fig. S9A; Additional file 5: Table S4). These two conditions also showed the highest number of transcripts with a significant 5’ shift (more negative polarity score) in ribosome density (Δpolarity < ** − **0.15; *p* < 0.05), followed by Ile, fewer under double starvation, and almost none under Leu (Fig. 4D). Transcripts affected by Val and triple starvation largely overlapped, whereas those altered by Ile were mostly distinct (Fig. 4E). Notably, in transcripts with a stronger 5’ polarity under Ile or Val starvation, the 5′ CDS was enriched for codons matching the limiting amino acid (Fig. 4F). Under triple starvation, only valine codons were enriched in 5′ regions of polarized transcripts, while no codon enrichment was seen under Leu or double starvation, consistent with minimal codon-specific effects. Interestingly, under Ile starvation, the analysis shows a significant enrichment of the Ile codon AUC, alongside a slight underrepresentation of both the Leu codon CUU and the Val codon GUU, likely due to specific transcript composition or codon co-occurrence biases. An illustrative example is *Cbx1*, a transcript with a large polarity shift under Val and triple starvation, containing multiple valine codons in its 5′ region, precisely where the ribosomes piled up (Fig. 4G). Similarly, *Hint1*, affected by Ile starvation, has isoleucine codons near its start codon in regions with increased ribosome accumulations (Fig. 4H). Of note, although *Hint1* contains an early valine codon, no clear signal increase was detected at this site. The overrepresentation of the mentioned codons was specific to the 5′ CDS regions of polarized transcripts; the total CDS of these transcripts did not show elevated frequencies of the limiting amino acid’s codons (Additional file 1: Fig. S9B).

The observed enrichment of Val codons in the 5′ regions of polarized transcripts supports the interpretation that early delays in elongation contribute to the RPF shift. In contrast, no such enrichment is observed for Leu starvation, reinforcing the idea that Leu-induced polarity is not driven by stalling at Leu codons, but likely a result of general stress responses. Moreover, the results suggest that BCAA starvation, especially Val starvation, can alter ribosome distribution along transcripts, with distinct codon- and condition-specific effects on elongation, leading to stalled ribosome progression and the accumulation of ribosomes in upstream regions.

### Non-uniformly distributed codons create elongation bottlenecks

**Fig. 5:**
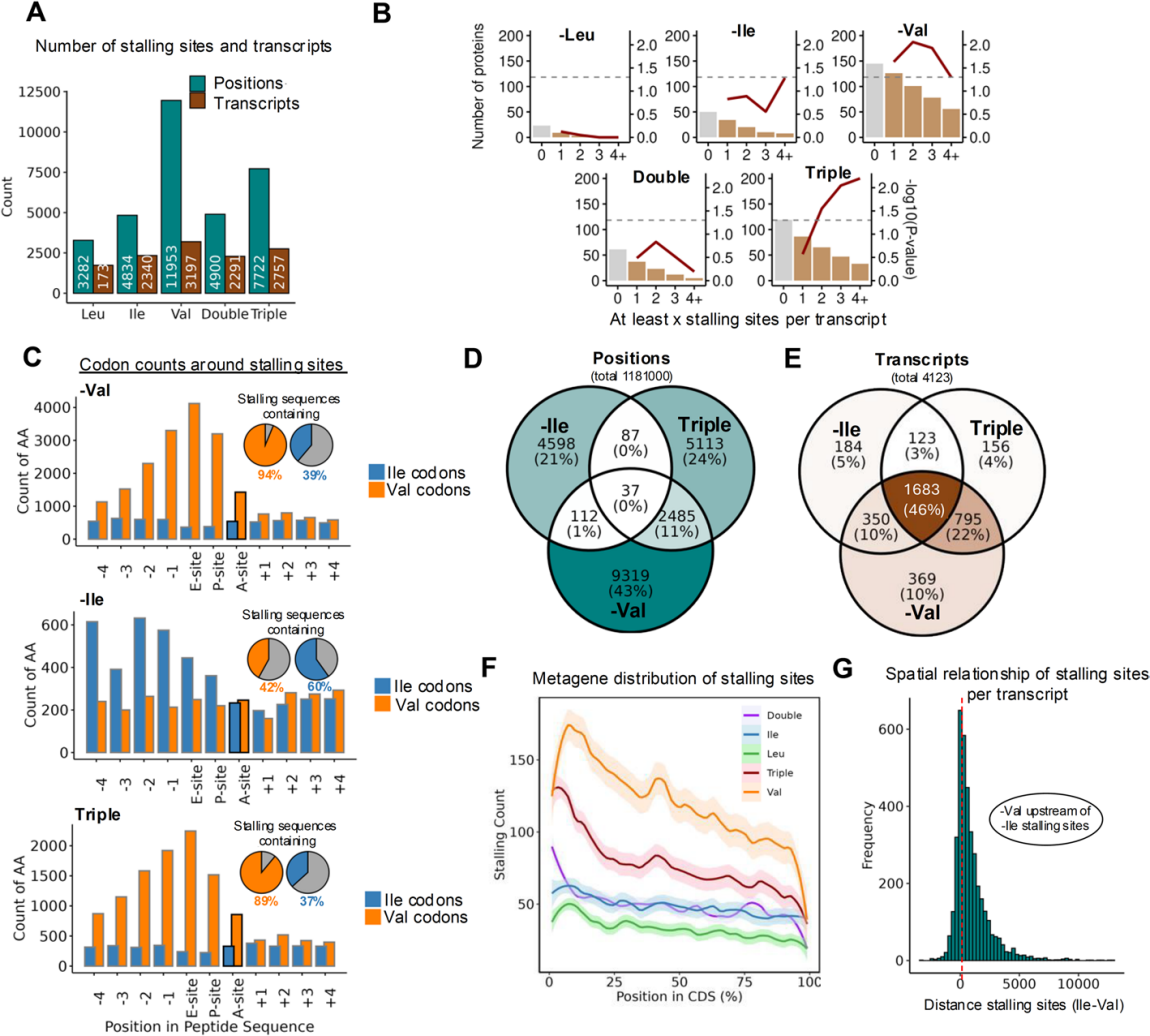
Non-uniformly distributed codons create elongation bottlenecks.** A** Bar plots showing the number of extracted stalling sites (positions) and the corresponding transcripts harboring stalling sites across different starvations. Peaks were defined based on the following criteria: log_2_FC(RPM) > 2 and log_2_FC(Norm_RPM) > 2 and FC_relative_to_gene_mean > 1. Peaks that met these thresholds and showed statistically significant differences (*p* < 0.05) between conditions were considered robust. **B** Number of significantly downregulated proteins (log₂FC < − 0.26, adjusted *p* < 0.01) that also exhibit at least the indicated number of stalling sites. The red line shows the statistical enrichment of overlap between stalling and downregulation, calculated using a hypergeometric test and plotted as − log₁₀(*p*-value). The dashed horizontal line marks the significance threshold (*p* = 0.05). The first bar (0) represents the total number of downregulated proteins per condition, irrespective of stalling. **C** Count graphs depicting the valine and isoleucine codon counts at indicated positions around the extracted stalling sites in the annotated starvation condition. The region surrounding each stalling site was extracted and the amount of Val or Ile codons was summed at each position; accompanying pie charts indicate, in percentage, the proportion of extracted stalling sequences that contain an Ile or Val codon versus those that do not. **D,E** Venn diagrams displaying the overlap of identified **D** stalling positions and **E** transcripts that harbor stalling sites in Ile, Val, and Triple starvation. **F** Metagene distribution of the extracted stalling sites across the CDS in all starvation conditions. **G** Spatial analysis of stalling sites on the 1683 transcripts harboring stalling sites in Ile, Triple, and Val starvation conditions. Per transcript, the distance between the first stalling site caused by -Ile starvation and the first stalling site caused by -Val starvation was calculated, revealing that under Val starvation the stalling occurs further upstream on the transcript

Despite the 5′-enriched ribosome accumulation under Val and triple starvation, it remains puzzling why cells upon triple starvation fail to show Ile-starvation-specific stalling, which were observed under single Ile starvation. As the metagene profiles did not pinpoint the exact codons along individual transcripts at which ribosomes paused, we proceeded to identify these sites more precisely by systematically mapping ribosome stalling events by peak-calling (Additional file 6: Table S5). This approach revealed strong differences in the frequency and distribution of stalling events across the different starvations: Val starvation caused the highest number of stalling events (11,953 sites across 3197 genes), which exceeded those observed under Leu, Ile, or double starvation (Fig. 5A; Additional file 1: Fig. S10A-B).

Intriguingly, cross-referencing transcripts containing identified stalling sites with the proteomic data revealed that the majority of proteins downregulated under Val starvation (see Fig. 3) harbored at least one stalling site (86.9%), indicating a possible link between the stalling sites and protein downregulation. Triple starvation also showed a high proportion of overlap (72.3%), but did not reach statistical significance. Other conditions showed weaker associations (-Ile: 68.0%; Double: 60.7%), while Leu starvation displayed the lowest overlap (39.1%) (Fig. 5B, Additional file 1: Fig. S10C), consistent with its minimal codon-specific stalling. Interestingly though, stratifying transcripts by the number of stalling sites revealed significantly stronger associations with downregulated proteins (Fig. 5B). Under Val and triple starvation, proteins that were downregulated were significantly enriched among transcripts with multiple stalling sites, an effect not observed under leucine, isoleucine, or double starvation (Fig. 5B). While the number of such transcripts and proteins dropped markedly in Leu and Double conditions, they remained relatively abundant in Val and Triple (Supplemental Fig. 10B). In addition, we observed that many downregulated proteins with associated stalling sites did not exhibit a net decrease in RPF signal in the Ribo-seq, as would be expected from predominating translation initiation defects (Supplemental Fig. 10C). Notably, RPF coverage is influenced by both initiation rates (with inhibition decreasing RPFs) and elongation dynamics, such as localized stalling (which can increase RPFs at specific positions). Consequently, transcripts affected by both impaired initiation and elongation may show little or no net change in total ribosome occupancy, thus, and a contribution of initiation changes to reduced protein output cannot be excluded. These findings highlight a potential contribution of elongation defects to the reduced protein output, particularly under Val starvation.

We focused our downstream analyses on the -Val, -Ile, and triple starvation conditions, as -Leu and double starvation induced only minor changes in ribosome dwell times and limited evidence of stalling. Both -Val and triple starvation consistently exhibited robust and highly similar stalling patterns across all analyses, whereas -Ile starvation displayed a distinct, non-additive profile with unique codon-level effects. To further investigate the basis of these non-additive responses, we examined the local sequence context surrounding the stalling sites identified under each condition. Under Val and triple starvation, we found that valine codons were strongly enriched directly at or just upstream of the identified stalling positions (A-site), whereas isoleucine codons were scarce and uniformly distributed (Fig. 5C). The pattern was reversed in cells upon Ile starvation, where isoleucine codons were abundant at or in close proximity to the stalling sites and valine codons were rarely seen (Fig. 5C). Of note, in cases where valine or isoleucine codons were present just upstream (rather than at) the stalling position, we noted a strong bias for GAG (E), GAA (E), GAU (D), GAC (D), AAG (K), CAG (Q), GUG (V) and GGA (G) (Val starvation) and AAC (N), GAC (D), CUG (L), GAG (E), GCC (A), CAG (Q), GAA (E), and AAG (K) (Ile starvation) at the stalling site (Additional file 1: Fig. S10D), codons known to be the slowest in the control condition (Additional file 1: Fig. S5H).

Consistent with the previous analysis, Val and triple starvation showed a substantial overlap in their stalling sites (2522 positions, *p* < 2.2 × 10^−16^), whereas few Ile-starvation-specific stalling positions persisted in triple starvation (Fig. 5D; 149 positions, *p* = 1.77 × 10^−52^). Nonetheless, when we examined entire transcripts rather than single positions, many transcripts that exhibited isoleucine-related stalling under Ile starvation also stalled under triple starvation (1806 transcripts, *p* = 1.78 × 10^−58^), but at different sites along the CDS (Fig. 5E). This finding is particularly intriguing, as it suggests that while Ile-starvation-specific stalling sites may shift under triple starvation, the overall tendency of these transcripts to stall remains. Given this dynamic redistribution, we focused on the positional patterns of stalling sites along the CDS and observed that under Val and triple starvation, stalling events tended to cluster toward the 5′ end of the CDS, whereas in the other starvations, stalling sites appeared more evenly distributed (Fig. 5F). Interestingly, these positional preferences mirrored the natural codon usage biases: We noticed a transcriptome-wide overrepresentation of valine codons in the early CDS, whereas isoleucine codons were comparatively more abundant toward the 3′ end (Additional file 1: Fig. S11A-B). This observation gives rise to a potential explanation as to why triple starvation stalling rarely targeted isoleucine sites: under triple starvation, an early stall of ribosomes on valine codons may create a bottleneck that may prevent or delay ribosomes from reaching downstream isoleucine codons (potentially owing to terminal stalling), thereby reducing the frequency of Ile-starvation-specific stalling. Thus, Ile-specific stalling sites are largely lost under triple starvation, while the same transcripts still tend to exhibit stalling, now primarily at upstream Val sites (Fig. 5D,E). Moreover, this concept was supported by detailed transcript-level analyses revealing that, in transcripts containing both Val- and Ile-starvation-specific stalling sites in their respective single starvation, the Val-starvation-specific stalling sites tend to lie upstream of the Ile-starvation-specific site (Fig. 5G). Taken together, these observations suggest that Val starvation establishes a translation bottleneck early in elongation, ultimately impacting downstream ribosome occupancy and overall protein output.

### Differential tRNA charging patterns reveal a mechanistic basis for codon-specific stalling under BCAA starvation

**Fig. 6:**
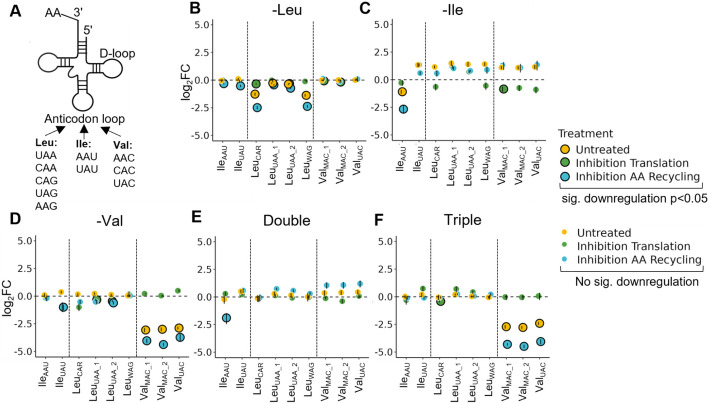
Differential tRNA charging patterns reveal a mechanistic basis for codon-specific stalling under BCAA starvation. **A** Schematic representation of the different tRNA isoacceptors for Leu, Ile, and Val. **B–F** Differential charging of tRNA isoacceptors under the indicated starvation conditions. Yellow indicates starved cells without treatment. Green represents cells treated with 100 µg/mL cycloheximide (CHX) in the last 30 min of starvation to inhibit translation. Blue represents cells treated with 10 µM MG132 and 160 nM Bafilomycin A1 to inhibit proteasomal and autophagic degradation (amino acid recycling inhibition). Certain tRNA isodecoders could not be measured separately and are therefore represented with IUPAC ambiguity codes: W (A or U), R (A or G), and M (A or C). Large outlined dots indicate significant downregulation (*p* < 0.05). Sample sizes: untreated (*n* = 5), translation and AA recycling inhibition (*n* = 2). Displayed is the mean of the replicates with the SEM indicated as error bars

Given our observations that early ribosome stalling under Val and triple starvation correlates with an overrepresentation of valine codons, we next investigated whether the charging levels of tRNA isoacceptors under these conditions fully reflect the apparent elongation bottleneck. If valine tRNAs become depleted more quickly than the other tRNAs under the triple starvation, this could explain why ribosomes stall on valine-rich regions near the 5′ end of transcripts and fail to progress to downstream codons. Moreover, differences in the charging status of tRNA isoacceptors could underlie the codon-dependent stalling patterns observed under isoleucine starvation (Fig. 1). To test this, we systematically measured the charging levels of tRNA isoacceptors for leucine, isoleucine, and valine (Fig. 6A) in each starvation condition using periodate oxidation and tRNA isoacceptor-specific RT-qPCR (adapted from [54]; see Methods).

Aligning with our stalling data, Val and triple starvation both resulted in a significant decrease of all charged tRNA^Val^ isoacceptors (Fig. 6D, F; yellow dots), while the triple starvation did not reduce charging for tRNA^Ile^ or tRNA^Leu^ (Fig. 6F; yellow dots). By contrast, individual Leu starvation selectively reduced the charging of tRNA^Leu^_CAR_ and tRNA^Leu^_WAG_ (while sparing tRNA^Leu^_UAA_) (Fig. 6B; yellow dots). Similarly, upon Ile starvation, only tRNA^Ile^_AAU_ lost charging, whereas tRNA^Ile^_UAU_ did not (Fig. 6C; yellow dots). Upon double starvation we could not observe a charging loss of any of the BCAA tRNAs (Fig. 6E; yellow dots). These isoacceptor-specific patterns correlate largely with the particular subsets of leucine and isoleucine codons that stalled (Fig. 1A). Of note, total tRNA levels remained largely unchanged across all starvation conditions (Additional file 1: Fig. S12A-E), suggesting that observed differences in charged tRNAs reflect genuine shifts in aminoacylation rather than alterations in overall tRNA abundance.

To clarify whether active translation directly consumes these aminoacyl-tRNA pools leading to accumulation of uncharged tRNAs, we treated cells with CHX for the final 30 min of deprivation. CHX blocks ribosome translocation and thus ongoing elongation. Notably, CHX rescued the depletion of tRNA charging in each condition (Fig. 6B–F; green dots), suggesting that, when the ribosomes are not actively translating, aminoacyl-tRNA synthetases can adequately charge tRNAs even in the face of limited amino acids. Lastly, we probed whether amino acid recycling via autophagy and proteasomal degradation affected tRNA charging and would lead to the accumulation of the uncharged version of additional tRNA isoacceptors. Inhibiting these pathways upon triple starvation did not cause a loss of any tRNA^Ile^ isoacceptor charging, but it did intensify the charging loss of tRNA^Val^ isoacceptors (Fig. 6F; blue dots). This aligns with our model that, upon triple starvation, ribosomes stall at valine codons near the transcript 5′ end, which can compromise their progression to isoleucine codons (e.g., in the event of terminal stalling at valine codons). As a consequence, the demand for charged tRNA^Ile^ would be reduced and blocking autophagy and proteasomes would not exacerbate charged tRNA^Ile^ depletion. By contrast, charged tRNA^Val^ are used immediately and become more depleted when proteolytic recycling is cut off.

Thus, tRNA isoacceptor charging dynamics closely reflect the codon usage and ribosome stalling patterns observed under BCAA starvation. Our data strengthen the link between codon bias, localized tRNA shortages, and the translational blocks that arise under nutrient-limited conditions.

## Discussion

Cells dynamically regulate translation in response to nutrient availability, ensuring adaptive protein synthesis under stress conditions. In this study, we present a comprehensive analysis of how cells modulate translation in response to BCAA starvation. By combining data from RNA-seq, Ribo-seq, quantitative proteomics, and tRNA charging assays, we revealed codon-specific ribosome stalling events triggered by the starvation of leucine, isoleucine, and valine. These events are associated with altered tRNA charging levels, activation of stress response pathways, and show codon positional effects that influence ribosome stalling.

### tRNA charging and codon-specific stalling

Our findings reveal that tRNA charging tightly correlates with the observed stalling patterns (Additional file 1: Fig. S12F), providing a mechanistic basis for the codon-specific translational effects. For valine, all measured tRNA^Val^ isoacceptors were strongly uncharged under both Val and triple starvation (Fig. 6). This directly correlates with a consistent increase in DTs for all Val codons (Fig. 1A). In the case of Ile starvation, only tRNA^Ile^_AAU_, which decodes AUU and AUC, showed a significant deacylation. Conversely, tRNA^Ile^_UAU_, which decodes AUA, remained charged (Fig. 6). This differential charging aligns perfectly with the observed increase in DTs for AUU and AUC codons, while AUA codons did not exhibit this effect (Fig. 1B, Additional file 1: Fig. S2A). The distinct behavior of the AUA codon might be due to a lower sensitivity of the specific tRNA^Ile^_UAU_ isoacceptor to starvation, a phenomenon supported by our tRNA charging data and previous studies [31, 63]. Furthermore, our spatial analysis (Additional file 1: Fig. S11B) confirmed that AUA codons tend to occur downstream of AUU and AUC codons within transcripts, which could eventually further reduce their stalling potential. Intriguingly, for leucine starvation, increased DT was observed only for CUU (Fig. 1A), which can be decoded by tRNA^Leu^_AAG_, and our charging assays indicate that this isoacceptor becomes slightly deacylated during leucine starvation. In contrast, tRNA^Leu^_UAA_, which decodes UUA and UUG via wobble, remains fully charged. Mild charging loss was observed for the Leu isoacceptors (tRNA^Leu^_AAG|UAG_, tRNA^Leu^_CAA|CAG_) that decode the other Leu codons which did not show significant dwell time increases. Importantly, as these tRNA isoacceptor pairs cannot be individually distinguished in our assay, it is possible that only one isoacceptor of each pair becomes uncharged, so that the other tRNA is still available to decode the corresponding codon. However, the overall effects on both DT and tRNA charging appeared very mild under Leu starvation. This observation aligns with early studies in *E. coli*, which predicted and later confirmed that when a single amino acid becomes limiting, certain tRNA isoacceptors lose their charging much faster than others, depending on their abundance and codon frequencies [31, 32]. Moreover, selective tRNA charging under stress in mammalian cells has been shown to modulate translation rates between stress-responsive and growth-associated transcripts [63]. Our data also indicate that a specific tRNA isoacceptor does not always become uncharged when its cognate amino acid is starved (i.e., triple starvation). Even under complete amino acid starvation, only a subset of tRNAs lose charging [54], suggesting that tRNA charging dynamics are governed by a balance between supply and demand. This redistribution of available translational resources allows ribosomes and tRNAs to be repurposed for the translation of stress-response transcripts [31, 32]. For instance, under double starvation (-Leu/-Ile), we observe the strongest mTORC1 inhibition (Fig. 2) and also the least dwell time modulation, even lower than in -Leu alone. This is consistent with the notion that initiation repression dominates in such conditions. Accordingly, no tRNA uncharging was detected for Leu or Ile isoacceptors under double deprivation (Fig. 6E), including those that were mildly affected in the single-starvation conditions. This is likely due to reduced initiation, which lowers the overall demand for charged tRNAs [43].

### Adaptive fidelity mechanisms and RNA modifications

Beyond direct tRNA availability, the translational response to BCAA starvation is further modulated by adaptive fidelity mechanisms. For instance, cells can allow amino acid misincorporation, such as the misacetylation of tRNA^Ile^ with valine by isoleucyl-tRNA synthetase (IARS1) under Ile starvation [64] or leucine misincorporation at phenylalanine codons under Phe starvation [65], a strategy now seen as an adaptive response rather than purely deleterious [66, 67]. In addition, amino acid starvation can also induce ribosomal frameshifting, leading to the production of aberrant proteins with potential implications for cellular functions [30, 68–70], or ribosomes may slide over codons and resume translation downstream [71].

Moreover, our proteomic data (Additional file 4: Table S3) suggest the involvement of RNA modifications, showing differential expression of OSGEP and MOCS3, enzymes crucial for tRNA modifications (t^6^A and mcm^5^s^2^U respectively) that ensure decoding accuracy [72–76]. Defects in these modifications can increase translational errors and activate stress response pathways, such as the GCN4-dependent expression of general amino acid control (GAAC) genes, even in the absence of amino acid starvation [77]. Similarly, our RNA-seq analysis revealed, particularly under Ile starvation, an enrichment of transcripts involved in the m^6^A pathway (Additional file 1: Fig. S6D). m^6^A has been shown to be dynamically regulated under stress and to regulate the ATF4 expression and autophagy induction under amino acid starvation [78, 79] and to influence translation elongation by altering the decoding kinetics by tRNAs and results in occurrence of ribosome stalling [80, 81].

### Codon positional effects and bottleneck phenomenon

Our analysis highlights that codon positional context can significantly modulate ribosome stalling during nutrient stress. It is well established that codon usage along mRNAs is not random but follows distinct positional patterns. Codon usage biases at the 5′ CDS region, enriched with rare or “suboptimal” codons, regulate ribosome flow and co-translational folding [82–85]. Expanding on these insights, our findings demonstrate that under BCAA limiting conditions, valine codon enrichment at the 5′ end of transcripts (Additional file 1: Fig. S11A) can lead to increased ribosome density and early ribosome stalling events. These stalling events possibly create a bottleneck that prevents ribosomes from reaching downstream regions of the mRNA, potentially masking other stalling sites further along the transcript (e.g., on Ile or Leu codons). Alternatively, even if ribosomes do not stall terminally, they might transiently slow down at these early valine codons and reduce the local, immediate demand for amino acids like isoleucine. Our finding that translation inhibition rapidly restores charged tRNA pools across all starvations (Fig. 6) supports this model: A local slowdown of translation at valine codons may allow aminoacyl-tRNA synthetases additional time to recharge Ile-tRNAs before the ribosome encounters Ile codons downstream, ultimately reducing stalling at downstream Ile codons during triple starvation.

We expect this positional effect to be potentially relevant for combinations of amino acids in which one amino acid has considerable enrichment near the 5′ end of coding sequences, coupled with starvation-sensitive tRNA isoacceptors, while the other does not. In our case, valine appears to meet these criteria (Additional file 1: Fig. S11A and Fig. 6). To explore the generalizability of this model, we have included a transcriptome-wide analysis of codon position biases in mouse for all codons (Additional file 1: Fig. S11A-C), which may serve as a basis to identify additional candidate codons for future studies. Based on existing predictions of tRNA deacylation in bacteria [31], amino acids such as phenylalanine and glutamine, whose tRNAs are expected to be highly deacylated and whose codons show some 5' enrichment (Additional file 1: Fig. S11C), could be promising candidates for testing this model. These could be tested in combination with amino acids whose tRNAs are expected to remain partially charged or whose codons are less frequent at the start of the CDS. Amino acid pairs like alanine vs. histidine also present interesting profiles for further investigation (Additional file 1: Fig. S11C). Reporter assays such as dual-fluorescence systems, potentially in a high-throughput screening format, may provide powerful ways to test the generalized bottleneck model and identify specific amino acid pairs.

### Elongation defects vs. initiation repression

While BCAA starvation broadly suppresses translation initiation, our data indicate that elongation defects contribute in codon- and condition-specific ways. Although ribosome accumulation at the 5′ ends of CDSs was observed across all BCAA starvation conditions (Fig. 4A), only Val, Ile, and Triple starvation produced a pronounced codon-specific effect at these early positions. In contrast, Leu and Double starvation resulted in a much weaker polarity shift (Fig. 4C) and showed no enrichment for Leu codons in the early CDS of polarized transcripts (Fig. 4E), suggesting a codon-independent redistribution of ribosomes potentially linked to general stress responses. The polysome profiles (Fig. 3F–H) generally indicate repressed initiation, but the manifestation of elongation defects varies. For valine starvation, strong, localized stalling at 5′-enriched Val codons (Fig. 5F) possibly acts as an early bottleneck, restricting overall ribosome movement and preventing polysome build-up, despite evident stalling. This may explain why strong stalling does not always correlate with polysome accumulation. Conversely, isoleucine codon stalling occurs more distally along the CDS (Fig. 5F), allowing more ribosomes to load and accumulate, leading to a more detectable polysome signal (Fig. 3F, H). It is also worth noting that the variability in Ribo-seq replicates for isoleucine starvation was slightly higher (Additional file 1: Fig. S6B), which might have reduced the sensitivity of stalling site detection and led to an underestimation of Ile stalling sites compared to valine.

We do not currently understand why the maximum pattern of valine or isoleucine dwell times does not sharply peak at the A-site but instead appears as a “smear” of elevated dwell times extending ~ 5 AA upstream, a property also observed in our reanalysis of previous starvation data (Additional file 1: Fig. S2). We consider technical reasons unlikely, as our Ribo-seq protocol avoids CHX pretreatment of live cells and therefore argues against drug-induced ribosome displacement as the cause for this pattern. Alternatively, the effect may be rooted in starvation- or stress-induced modification of the ribosome (e.g., posttranslational modifications such as ubiquitination) that transiently alter the elongation rate of codons following the initial stalling, especially at codons that are already slow on average.

### Future directions

Although we identified ribosomal stalling sites on transcripts with simultaneous decreases in protein synthesis as a key feature of valine deprivation, our study does not directly measure the downstream consequences of the stalled translation on protein degradation, cellular proteostasis, or mRNA stability. Recent work has shown that persistent ribosome stalling can activate ribosome-associated quality control (RQC) pathways, which facilitate nascent chain degradation and can also trigger targeted decay of the associated mRNA [86, 87]. Moreover, ribotoxic stress responses can be initiated by ribosome collisions or prolonged stalling, leading to activation of stress kinases such as JNK and influencing broader proteostatic programs [88, 89]. These mechanisms underscore how terminal stalling or slowed elongation can have far-reaching consequences for both the protein and RNA components of the translational apparatus. However, it remains unclear whether the observed stalling in our BCAA starvations induces terminal stalling, or whether a deceleration of elongation without termination, may cause a differential engagement of RQC and ribotoxic stress pathways. Future studies employing disome (collided-ribosome) sequencing approaches and specialized reporter assays, combined with global proteostasis analyses and mRNA stability measurements, will be necessary to disentangle the precise nature of ribosome slowdown and its direct impact on protein turnover and quality control mechanisms. It would also be valuable to investigate whether ribosomes undergo modifications during starvation that alter their decoding properties, which could act as a complementary mechanism to the queuing effects we observed. Additionally, examining other combinatorial BCAA starvation conditions (e.g., Leu/Val or Ile/Val), which was a limitation of the current study, would be valuable to further explore these complex translational dynamics and codon-level differences.

Furthermore, understanding the physiological relevance of the valine-dependent bottleneck observed under broad BCAA scarcity is a critical area for future investigation. While complete starvation of all three BCAAs is rare, such conditions are physiologically relevant in contexts like severe malnutrition, cachectic states associated with chronic diseases, or in specific therapeutic interventions such as specialized diets for Maple Syrup Urine Disease (MSUD) management [90, 91]. Further research is needed to determine how cells might use this valine-specific translational checkpoint to rapidly conserve resources and potentially prioritize the synthesis of essential stress-response proteins during starvation. Exploring the precise gene sets that are differentially regulated under these conditions would provide deeper insights into this adaptive mechanism.

## Conclusions

Our integrated analysis reveals a significant codon-specific impact of ribosome stalling on translation during BCAA starvation, which varies across single and combined deprivation conditions. We demonstrate that these effects are not merely a consequence of amino acid availability but are shaped by a complex interplay between tRNA isoacceptor charging, codon identity, and importantly, the positional distribution of codons along mRNAs. The early enrichment of valine codons in coding sequences appears to establish a translation bottleneck under valine and triple starvation, which limits ribosome progression and alters downstream elongation dynamics. This positional stalling selectively modulates translation efficiency and contributes to reduced protein output. Our findings suggest that these effects are modulated by the positional distribution of codons along the mRNA, potentially creating a novel bottleneck phenomenon that underscores an underappreciated regulatory mechanism in cellular adaptation to nutrient stress.

## Methods

### Cell culture and amino acid starvation

NIH3T3 mouse fibroblast cells were cultured in DMEM (high glucose, pyruvate; Gibco) supplemented with 10% FBS (qualified, USDA-approved regions; Gibco), and 100 U/ml penicillin–streptomycin (Gibco). Cells were maintained at 37 °C in a humidified incubator with 5% CO₂. Amino acid starvation experiments were conducted using custom DMEM/F-12 media (Gibco; SKU11320 modified) supplemented with 10% dialyzed FBS (Gibco) and lacking specific BCAAs—leucine, isoleucine, and valine—as well as asparagine and aspartate. L-Asparagine monohydrate (7.5 mg/l) and L-aspartic acid (6.65 mg/l) were added back to all media. Leucine (59.05 mg/l), isoleucine (54.47 mg/l), and valine (52.85 mg/l) were supplemented in specific combinations to create the following media: Complete medium (containing all amino acids at standard concentrations), Leucine starvation (medium lacking leucine), Isoleucine starvation (medium lacking isoleucine), Valine starvation (medium lacking valine), Double starvation (medium lacking both leucine and isoleucine), Triple starvation (medium lacking leucine, isoleucine, and valine). The amino acids were purchased in powdered form from Sigma-Aldrich. Cells were washed once with 1xPBS (Gibco) before switching to the respective starvation medium for 6 h. The NIH3T3 cell line was not independently authenticated. Cells were routinely checked for mycoplasma contamination.

### Ribo-seq and RNA-seq

Cells were washed with cold PBS (Gibco) supplemented with 100 µg/ml cycloheximide (Sigma-Aldrich), lysed in 200 µL polysome lysis buffer (20 mM Tris–HCl pH 7.4, 150 mM NaCl, 5 mM MgCl_2_, 1% Triton X-100, 5 mM DTT, 100 µg/ml CHX, 25 U/mL TURBO DNase (Invitrogen), 10 U/µL murine RNase inhibitor (NEB), and protease/phosphatase inhibitors (Sigma-Aldrich). Note: Cycloheximide (CHX) was added only during the cold PBS washes and in the lysis buffer, without any pretreatment of live cells, in order to minimize ribosome run-off while avoiding CHX-induced elongation artifacts described in previous studies [44]. Cells were scraped down and transferred into microcentrifuge tubes. After incubation on ice for 10 min, lysates were passed through a 26G needle 10 times and centrifuged at 20,000* g* for 10 min at 4 °C. Supernatants were collected and quantified by NanoDrop. Ribosome-protected fragments (RPFs) were obtained by adding 43 U/OD260 RNase I (Invitrogen) at 24 °C for 45 min. The reaction was stopped with 5 µL SUPERase In RNase inhibitor (Invitrogen) and placed on ice. Digested samples were purified using Microspin S-400 HR columns (Cytiva) equilibrated with polysome buffer (20 mM Tris–HCl pH 7.4, 150 mM NaCl, 5 mM MgCl₂, 1 mM DTT, and 100 µg/ml cycloheximide) for 2 min at 2400 rpm, followed by phenol–chloroform extraction and ethanol precipitation. Precipitated RNA was resuspended in water and quantified. For size selection, 15% Novex TBE-Urea polyacrylamide gels (Invitrogen) were pre-run for 20 min at 200 V. Samples were mixed with Novex TBE-Urea sample buffer (Invitrogen), heated at 90 °C for 2 min, and loaded onto the gels, alongside RNA size markers (NEB). Gels were run at 170 V for approximately 70 min and stained with SYBR Gold gel stain (Invitrogen). Gels were excised under blue light, and fragments between 25 and 36 nucleotides were purified. To extract the RNA from gel fragments, a 0.5-mL microcentrifuge tube was pierced at the bottom with an 18G needle, the cap was removed, and the tube was placed inside a 1.5-ml microcentrifuge tube. The gel fragment was placed inside the prepared 0.5-ml tube and centrifuged for 2 min at full speed to collect the gel debris in the lower tube. Three hundred sixty microliters of water was added to the gel debris and incubated at 70 °C for 10 min. The resulting gel slurry was transferred into a microfuge tube spin filter (Corning Costar Spin-X) and centrifuged for 3 min at full speed. The filtrate was collected in a new tube, and 40 µL of 3 M sodium acetate, pH 4.5 was added. Subsequently, 1.5 µl of GlycoBlue (Invitrogen) was added, followed by 700 µL of isopropanol. The mixture was incubated overnight at − 20 °C for precipitation. RNA was collected by centrifugation, pellets were washed with 80% ethanol, dried, and resuspended in water for further processing. rRNA was depleted by hybridizing RNA samples with complementary biotinylated oligonucleotides (Additional file 7: Table S6) and hybridized rRNA was removed using MyOne Streptavidin C1 Dynabeads (Invitrogen). Samples were purified using a RNA Clean & Concentrator kit (Zymo). End repair was performed by incubating RNA with T4 PNK (NEB) at 37 °C for 1 h, followed by ATP addition and another hour of incubation. Clean-up was performed using the RNA Clean & Concentrator kit (Zymo). Libraries were prepared using the NEXTflex Small RNA-Seq Kit v3 (Revvity) according to the manufacturer’s protocol. RNA from the same experimental conditions was used for RNA-seq analysis. For this, RNA was extracted from the polysome lysates before RNase I digestion by phenol–chloroform extraction and ethanol precipitation. Total RNA was submitted to the EPFL Gene Expression Core Facility (GECF) for mRNA library preparation using NEBNext Ultra II Directional RNA Library Prep with PolyA selection. RNA-seq libraries were sequenced on a Novaseq6000 generating paired-end (50/50) reads, and Ribo-seq libraries were sequenced on a Nextseq500 system generating single-end 75 reads.

### RNA-seq and Ribo-seq data processing

For RNA-seq, paired-end reads were aligned to the Ensembl mouse GRCm38 primary assembly reference genome using STAR (v2.7.11a) [45]. The corresponding Ensembl GTF annotation (GRCm38.100) was used to build the genome index, and gene-level counts were generated with the –quantMode GeneCounts option. Reads mapping in the sense orientation relative to annotated transcripts were retained. On average, approximately 51 million reads per sample were uniquely mapped and quantified.

For Ribo-seq, adapter sequences were trimmed from raw Ribo-seq reads using cutadapt [46] with the parameter -m 10 and the adapter sequence TGGAATTCTCGGGTGCCAAGG. Using an in-house Perl script, we then removed duplicate reads carrying identical sequences and UMIs (four random nucleotides at both 5′ and 3′ ends). The processed reads were first aligned to a combined mouse and human rRNA/tRNA reference (from UCSC genome browser) using STAR to remove and quantify rRNA/tRNA contaminants, then mapped to the same GRCm38 reference genome used for RNA-seq. To select ribosome-protected fragments, only reads between 25 and 35 nucleotides in length with a unique alignment ([NH] = = 1) were retained using samtools. Feature distribution was assessed with RSeQC read_distribution.py [47] using the mm10_GENCODE_vm25.bed file on the filtered BAM files. Finally, gene-level counts were primarily generated with htseq-count using the intersection-strict mode [48] from the same filtered BAM file, as well as the same GTF annotation used for the RNA-seq analysis. For a comparative analysis, an additional count matrix was generated including multi-mapped reads (htseq-count –nonunique all -a 0). This multimapping dataset is used in Additional file 1: Fig. S7 for comparison, while all other analyses are based on uniquely mapped reads.

Only protein-coding genes were retained for further analysis, and genes without gene symbol annotation or with fewer than 10 reads were removed. Differential gene analysis was performed in R using edgeR: Separate DGEList objects were created for RNA-seq and Ribo-seq datasets and normalized, followed by estimation of dispersion parameters using estimateDisp. A design matrix was constructed, and genewise quasi-likelihood negative binomial GLMs were fitted. Differential expression (DE) testing was performed using glmQLFTest for each contrast of interest. For each condition, DE results were extracted, and *p*-values were adjusted for multiple testing using the Benjamini–Hochberg procedure. Ribosome densities (RD) were calculated as the ratio of the RPM of Ribo-seq/RNA-seq.

Dwell time modeling: For ribosome dwell time inference and gene ribosome density profiles, the Ribo-seq de-duplicated reads were input to the Ribo-DT pipeline [49] using similar reference genomes as above. Parameters were set as follows: a lower bound read size (L1) of 26 nucleotides, an upper bound (L2) of 35 nucleotides, library strandness specified as “pos_neg,” and a filter threshold of 10 reads per gene. Single dwell time output table was used for all subsequent analysis. Intermediate “.count” Ribo-DT output files were used to generate ribosome density profiles. Mean DTs for each experimental condition were calculated by averaging triplicate values. Unless specified otherwise, codon-level DTs were filtered to include specific ribosomal positions ("−2", "−1", "E", "P", "A", "3", "4") and summarized as mean values per codon. For statistical analysis, codon-specific ANOVA models were applied to compare DTs across conditions, followed by post hoc Tukey’s Honest Significant Difference (Tukey HSD) tests to identify significant differences between experimental conditions and the control.

### Polysome profile generation and analysis

Polysome lysates were prepared as described above for Ribo-seq analysis. Sucrose gradients (5–50% in polysome buffer: 20 mM Tris–HCl pH 7.4, 150 mM NaCl, 5 mM MgCl₂, 1 mM DTT, and 100 µg/ml cycloheximide) were prepared using a gradient maker (Biocomp). Lysates were loaded onto the gradients and centrifuged at 32,000 rpm for 2.5 h at 4 °C in an ultracentrifuge (Beckman Coulter). Polysome profiles were generated by fractionating the gradients with continuous UV monitoring at 260 nm using an automated gradient fractionation system (Biocomp). The raw data were further processed with R: Raw polysome profiles were smoothed using moving average filters to reduce noise. For alignment, manually selected reference points representing key structural features of the profiles (anchor points; monosome and polysome peaks, and local maxima and minima) were defined for each sample. To standardize baseline values, each profile was shifted such that the overall minimum intensity was set to zero. Following this, the defined anchor points were used to perform a linear time axis interpolation to correct for run-to-run variation in migration patterns. After alignment, the intensity of each profile was normalized to its total area under the curve (AUC), enabling cross-sample comparisons, and AUC for polysome and monosome area was calculated. Polysome-to-monosome ratios (P/M) were calculated and values were further normalized to the control condition. Statistical analyses were performed using unpaired *t*-tests. All results are presented as mean ± SEM, with statistical significance indicated.

### Intracellular amino acid measurement

Cells were incubated in the respective starvation media for 6 h, as detailed above. After treatment, cells were washed twice with ice-cold phosphate-buffered saline (PBS) to remove extracellular amino acids. Plates were then flash-frozen on dry ice to preserve intracellular metabolites. Frozen samples were submitted to the Metabolomics Unit at the University of Lausanne (UNIL) for analysis. Amino acids were quantified using a stable isotope dilution LC–MS approach, following established protocols [50]. Each condition was analyzed in five biological replicates.

### Western blot analysis

Protein lysates were prepared by resuspending cell pellets in a hot SDS lysis buffer (2% SDS, 50 mM Tris–HCl, pH 7.5, 1 mM EDTA) and heating samples at 95 °C for 5 min. Afterwards, samples were centrifuged for 5 min at max speed and supernatant transferred to a new tube. Protein concentrations were measured using a NanoDrop spectrophotometer (Thermo Fisher Scientific). Protein lysates were mixed with NuPAGE LDS sample buffer (Invitrogen) and 50 mM DTT. Equal amounts of protein (8 µg per lane) were separated on 8–12% gradient SDS-PAGE gels (SurePAGE, Bis–Tris; GeneScript) and transferred onto PVDF membranes using the iBlot 2 Dry Blotting System with PVDF transfer stacks (Invitrogen). Membranes were blocked in 5% BSA in PBS-T (PBS with 0.1% Tween-20) for 1 h at room temperature. Primary antibody incubation was performed overnight at 4 °C in 5% BSA in PBS-T. After washing three times with PBS-T, membranes were incubated with HRP-conjugated secondary antibodies for 1 h at room temperature. Chemiluminescent signals were detected using the Fusion FX imaging system (Vilber). Protein band intensities were quantified using ImageJ software (NIH), and target protein levels were normalized to the corresponding loading control. Results represent the mean ± standard deviation from three independent biological replicates. Significance was determined by unpaired *t*-tests.

### RT-qPCR analysis

Total RNA was extracted using TRIzol reagent (Thermo Fisher Scientific) according to the manufacturer’s instructions. RNA concentration and purity were assessed using a NanoDrop spectrophotometer (Thermo Fisher Scientific). Complementary DNA (cDNA) synthesis was performed with 1 µg of RNA using M-MLV Reverse Transcriptase (Promega) and random hexamer primers in a 20 µL reaction. qPCR was carried out using a SYBR Green-based qPCR master mix (Promega) according to the manufacturer’s protocol on a QuantStudio 7 Real-Time PCR System (Applied Biosystems). Cycling conditions included an initial denaturation at 95 °C for 2 min, followed by 40 cycles of denaturation at 95 °C for 15 s and annealing/extension at 60 °C for 1 min. Relative gene expression levels were determined using the ΔΔCt method, normalizing target gene expression to Gapdh as a housekeeping gene. Data represent the mean ± standard deviation from biological replicates, with each sample analyzed in technical duplicates. Used primers can be found in the Additional file 7: Table S6.

### OPP incorporation assay

OPP (O-propargyl-puromycin) incorporation was used to assess protein synthesis following 6 h of different starvation conditions. Approximately 8000 cells per well were seeded into black 96-well plates with glass bottoms (Greiner Bio-One) the day before the assay. OPP was added directly to the culture medium at a final concentration of 25 µM (prepared from a 2.5 mM stock solution in DMSO, diluted 1:100) and incubated with cells for 20 min. After incubation, the medium was removed, and cells were washed twice with PBS. Cells were fixed with 4% PFA in PBS (ABCR) for 5 min at room temperature. Following fixation, cells were permeabilized with 0.1% Triton X-100 in PBS for 5 min and subsequently washed twice with PBS and once with PBS containing 3% BSA. Click chemistry was performed by incubating cells with 150 µl of reaction mix containing 1 × PBS, 500 µM CuSO₄, 2 µM 5-TAMRA-Azide (Jena Bioscience), and 50 mM L-ascorbic acid (Sigma Aldrich) for 45 min at room temperature. After incubation, cells were washed sequentially: once with 0.5 mM EDTA in PBS, twice with PBS containing 3% BSA, and finally with PBS containing Hoechst 33,342 (1:10,000 dilution, Thermo Scientific) for nuclear staining. Fluorescence images were acquired using the Operetta High-Content Imaging System (PerkinElmer). OPP incorporation and nuclear staining were quantified for individual cells using the Harmony software (PerkinElmer). Local background fluorescence was subtracted, and to account for autofluorescence, data from unstained samples were subtracted from all experimental conditions. Outliers were identified and removed using the interquartile range (IQR) method, excluding values outside 1.5 × IQR. Fluorescence intensities were further normalized to the mean of control samples. Pairwise comparisons to control samples were performed using Bonferroni-adjusted unpaired *t*-tests.

### TMT-based quantitative proteomics

Cells were seeded in 6-well plates 1 day prior to treatment. The medium was replaced with 2 ml of deprivation medium, and cells were incubated for 6 h. After incubation, cells were trypsinized, centrifuged, and washed once with PBS. The cell pellets were resuspended in 100 µL of lysis buffer containing 2% SDS, 50 mM Tris (pH 8.0), protease, and phosphatase inhibitors. Samples were boiled for 5 min and passed through a 26G needle 10 times for homogenization, followed by centrifugation at maximum speed for 5 min to remove debris. Protein concentrations were determined by BCA assay. Equal amounts of protein from each sample were processed for TMT-based quantitative proteomic analysis at the EPFL Proteomics Core Facility. Samples were digested by FASP (Filter Aided Sample Preparation) following the standard procedure with minor modifications [51]. Protein samples containing 20 µg were prepared in 2% SDS, 100 mM Tris–HCl pH 8.0 and deposited on conditioned filter devices (30 K). Devices were centrifuged at 10,000 × *g* for 30 min until complete dryness and then washed twice with 200 µL of urea solution (8 M Urea, 100 mM Tris–HCl pH 8.0). Reduction was performed on top of the filters with 100 µL of 10 mM TCEP in 8 M urea, 100 mM Tris–HCl pH 8.0 at 37 °C for 60 min with gentle shaking. Reduction solution was removed by extended centrifugation followed by two extra washing steps. Alkylation was then performed on top of the filters with 100 µL of 40 mM chloroacetamide in 8 M urea, 100 mM Tris–HCL pH 8.0 at 37 °C for 45 min light protected with gentle shaking. After removal of alkylation solution and two additional washing steps with 200 µL of urea solution, the filters were further washed twice with 200 µL of 5 mM Tris–HCl pH 8.0. Samples were then digested overnight on top of filters at 37 °C with 100 µL of proteolytic solution at 1/50 w/w enzyme-to-protein ratio using a combination of mass spectrometry grade trypsin and LysC supplemented with 10 mM CaCl_2_. Elution from filters of resulting peptides was achieved by extended centrifugation and two extra elutions were performed with 50 µL of 4% TFA. Samples were then desalted on SDB-RPS Empore StageTips using the standard protocol [52] and dried by vacuum centrifugation. Dried peptides were reconstituted in 10 µL 100 mM HEPES pH 8, and then, 5 µL of TMT solution (25 µg/µL in pure acetonitrile) was directly added. Labeling was achieved with the TMTproTM 18plex isobaric Mass Tagging Kit for 90 min at room temperature and reactions were then quenched with hydroxylamine to a final concentration of 0.4% (v/v) for 15 min. In order to avoid final extra normalization, a minor fraction of TMT-labeled samples were pooled at 1:1 ratio across all samples and a single LC-MS2 run was performed to ensure correct peptide mixing. Corrected mixing quantities of each TMT-labeled sample were calculated based on the control run and final combined samples were desalted using a 100-mg Sep-Pak tC18 cartridge according to provider recommendations and dried by vacuum centrifugation. Pooled samples were fractionated into 12 fractions by Agilent OFF-Gel 3100 system following the manufacturer’s instructions. The resulting fractions were desalted again using SDB-RPS Empore StageTips and dried by vacuum centrifugation. Each fraction was resuspended in 2% acetonitrile, 0.1% formic acid, and nano-flow separations were performed on an Ultimate 3000 RSLC UHPLC system online connected with an Orbitrap Fusion Lumos Tribrid Mass Spectrometer (ThermoFisher Scientific). Samples were first trapped on a capillary pre-column (Acclaim Pepmap C18, 3 µm-100 Å, 2 cm × 75 µm ID), followed by an analytical separation at 250 nl/min over a 150-min biphasic gradients through a 50-cm-long in-house packed capillary column (75 µm ID, ReproSil-Pur C18-AQ 1.9 µm silica beads, Dr. Maisch). The data was acquired through a Top Speed Data-Dependent acquisition mode using three seconds cycle time. First MS scans were acquired at a resolution of 120,000 (at 200 m/z), with the most intense parent ions selected and fragmented by High Energy collision Dissociation (HCD) with a Normalized Collision Energy (NCE) of 35% using an isolation window of 0.7 m/z. Fragmented ion scans were acquired with a resolution of 50,000 (at 200 m/z) and selected ions were then excluded for the following 60 s. Resulting Raw files were searched using multiple Database search engines (Sequest HT, Mascot, MS Amanda and MSFragger) in Proteome Discoverer (v. 2.5) against the Uniprot_Mouse_55286Sequences_LR2022_05 supplemented with classical MaxQuant contaminants. Enzyme specificity was set to trypsin with a maximum of two missed cleavages allowed. A 1% FDR cut-off was applied both at peptide and protein identification levels. For the database search, carbamidomethylation (C) and TMTpro tags (K and Peptide N termini) were set as fixed modifications while oxidation (M) was set as a variable one. Resulting text files were processed through in-house written R scripts (version 4.1.2). A first normalization step was applied based to the Sample Loading normalization [53]. Assuming that the total protein abundances were equal across the TMT channels, the reporter ion intensities of all spectra were summed and each channel was scaled according to this sum, so that the sum of reporter ion signals per channel equals the average of the signals across samples. A second normalization was applied using TMM (trimmed mean of M-values), and dispersion estimates were obtained using a generalized linear model (GLM) framework. Log_2_ fold changes and *p*-values were calculated using quasi-likelihood F-tests, with multiple testing correction applied using the Benjamini-Hochberg (BH) method. The final dataset includes log_2_FC values and adjusted *p*-values for each condition compared to the control. All conditions were analyzed in triplicates, except for the Double condition, which was analyzed in duplicates.

### Differential protein expression and protein half-lifes

Differentially expressed proteins were identified for all starvation conditions based on significance thresholds (*p* < 0.01) and |log_2_FC| cutoffs (> 0.26). For comparison, theoretical log_2_FC values after 6 h were estimated based on protein half-life assuming standard degradation kinetics, with a reduction in synthesis following the starvation (two scenarios are indicated as guides to the eye in Fig. 3J: synthesis decreasing to either zero or half). Protein half-lives in 3T3 cells were obtained from an external dataset [59] and assumed not to change during the 6-h starvation. One-way ANOVA followed by Tukey’s post hoc test was conducted to determine statistical differences in half-life distributions between up and downregulated protein groups.

### tRNA charging assay

The protocol was adapted from [54]. Cells were lysed in 500 µl TRIzol (Invitrogen) for 5 min at room temperature. After lysis, 100 µl chloroform was added, and samples were incubated on ice for 3 min. Following centrifugation (18,600 × *g*, 10 min, 4 °C), the aqueous phase was transferred to a new tube containing 250 µl isopropanol, mixed, and incubated at − 20 °C for 10 min. RNA was pelleted (18,600 × *g*, 15 min, 4 °C), washed with 80% cold ethanol, air-dried, and resuspended in 10 µL tRNA resuspension buffer (10 mM sodium acetate pH 4.5, 1 mM EDTA). For oxidation, 2 µg RNA was diluted in tRNA resuspension buffer (16.87 µL total volume) and incubated with 1 µL 0.2 M freshly prepared sodium periodate (NaIO₄) at room temperature for 20 min (control samples received 1 µL 0.2 M NaCl instead). The reaction was quenched with 2.75 µL 2.5 M glucose. After quenching, 1 µL *E. coli* spike RNA (4 ng/µL; Additional file 7: Table S6), 1 µL GlycoBlue (Invitrogen), and 1.3 µL 1 M NaCl were added. RNA was precipitated with 75 µL ice-cold 100% ethanol at − 20 °C for 30 min, pelleted (18,600 × *g*, 15 min, 4 °C), washed with 70% ethanol, air-dried, and resuspended in 100 µL 50 mM Tris–HCl pH 9.0. Deacylation was performed at 37 °C for 45 min, quenched with 100 µL tRNA quench buffer (50 mM sodium acetate pH 4.5, 100 mM NaCl), and precipitated overnight at − 20 °C with 540 µL ice-cold 100% ethanol. The RNA pellet was washed with 80% ethanol and dissolved in 5 µL water. For adaptor ligation, 800 ng RNA was mixed with 0.5 µL 10 µM tRNA adaptor (Additional file 7: Table S6), 1 µL 10 × T4 RNA ligase buffer (NEB), 0.4 µL 0.1 M DTT, 2.5 µL 50% PEG, 0.5 µL murine RNase inhibitor (NEB), and 0.25 µL T4 RNA Ligase 2, truncated KQ (NEB) in a final volume of 10 µL. The reaction was incubated at room temperature for 3 h, followed by overnight incubation at 4 °C. Reverse transcription was initiated by annealing 0.5 µL 5 µM RT primer (Additional file 7: Table S6) to 2 µL ligated RNA (65 °C for 5 min, then immediately chilled on ice). A master mix containing 0.8 µL 5 × SuperScript IV buffer (Invitrogen), 0.2 µL 10 mM dNTPs, 0.2 µL 0.1 M DTT, 0.2 µL murine RNase inhibitor, and 0.2 µL SuperScript IV enzyme (Invitrogen) was prepared, and 1.6 µL was added to the annealed RNA (final reaction volume: 4 µL). Reverse transcription was performed at 55 °C for 30 min, followed by inactivation at 80 °C for 10 min. The cDNA was diluted to 15 µL with water. qPCR was performed using tRNA-specific primers (Additional file 7: Table S6), and Ct values were normalized to the *E. coli* spike-in control. Experimental samples under starvation conditions were normalized to control samples grown in full medium. For inhibition studies, cells were treated during starvation with either Bafilomycin A1 (160 nM, Sigma Aldrich) and MG132 (10 µM; Sigma Aldrich) to inhibit amino acid recycling, or cycloheximide (100 µg/mL; Sigma Aldrich) for the last 30 min to block translation.

### Analysis of transcripts involved in mTORC1 and GCN2 pathways

To analyze the activation of specific stress response pathways, we utilized the log_2_FC datasets of the Ribo-seq and RNA-seq. The ATF4 target genes were identified as the top 150 targets from ChIP-Atlas [55], while the mTORC1-sensitive genes were selected as known TOP transcripts from Thoreen et al. [22]. Statistical significance between conditions was assessed using unpaired *t*-tests.

### Preparation of genome-wide codon count table

The CDSs of the mouse genome (Mus musculus, GRCm39) were extracted and processed using R. Codon frequencies were extracted by splitting sequences into triplets. Codon counts were calculated for each gene, and isoform-specific counts were averaged to obtain gene-level codon frequencies. The codon count table was normalized by dividing each codon count by the total codon count per gene, resulting in the fraction of codons per gene. The genes included in this table were further filtered for transcripts detected to be expressed in mouse NIH3T3 cells according to our RNA-seq and Ribo-seq experiments. Thus, this table includes the relative usage of each codon across all expressed genes in the mouse NIH3T3 cell genome and was used for the downstream analyses.

### Processing and analysis of position-specific Ribo-seq data

Raw count data were filtered and normalized to the library size (to obtain positional RPM) and further normalized by dividing each RPM by the respective total RPM of the transcript (Norm_RPM). Genes with low counts (mean total counts across all samples below 50) were excluded. Coverage filtering was applied by calculating the fraction of positions with non-zero counts for each gene. Genes with mean positional coverage below 30% were excluded, resulting in a set of 4123 transcripts.

For codon-specific ribosome density enrichments, codons of interest were identified within the CDS, and positional Ribo-seq data were extracted in a window of ± 50 nucleotides around each codon of interest. For each position, the relative Norm_RPM was computed as the ratio of experimental Norm_RPM to the control Norm_RPM baseline. Mean relative values across replicates were summarized for each codon and position.

For metagene profiles, the first and last 15 nt of each transcript were excluded from the analysis and positional RPMs were aligned relative to the start and end of the CDS. The normalized position was computed as a percentage of CDS length. Aggregated norm_RPM values were calculated across all transcripts for each condition and normalized position. To quantify ribosome accumulation at the 5′ and 3′ ends of transcripts, the summed norm_RPMs were calculated for two regions: the first 20% (5′ region), and last 20% (3′ region) of the CDS. These scores were then normalized to the control condition by calculating the ratio of each condition score to the mean control score per gene.

### Polarity score analysis

The polarity score was used to quantify the bias of ribosome density between the 5′ and 3′ regions of CDSs and calculated as previously described [56]. Positions within the CDS that are closer to the 5′ end will result in a negative contribution to the polarity score, while positions closer to the 3′ end will contribute positively. This means that a higher polarity score indicates greater ribosome density near the 3′ end of the CDS, while a lower or negative polarity score suggests more ribosomes near the 5′ end. The Δpolarity score was calculated for each transcript by subtracting the Ctrl score and significance assessed by unpaired *t*-test. Transcripts with a significant increase in ribosome occupancy in the 5′ region in comparison to the Ctrl condition (Δpolarity < − 0.15, *p* < 0.05) were extracted for further codon usage analysis. Codon frequencies were calculated for leucine, isoleucine, and valine codons in the first 20% of the CDS and normalized to the total codon count in that gene. Statistical comparisons were performed using unpaired BH-adjusted *t*-tests. The log_2_FC of codon usage was calculated relative to the control.

### Peak calling analysis

To identify positions and transcripts exhibiting ribosome stalling upon starvation, we computed for every transcript position the RPM and norm_RPM fold change relative to the control condition. Each position was also tested for having a higher RPM than the transcript mean. Statistical significance was assessed using unpaired *t*-tests. Peaks were defined based on the following criteria: log_2_FC(RPM) > 2, log_2_FC(Norm_RPM) > 2, and FC_relative_to_gene_mean > 1. Peaks that met all three thresholds and showed statistically significant differences (*p* < 0.05) between conditions were considered robust. To investigate the distribution of stalling sites along CDS, positions of upregulated stalling sites were mapped onto relative CDS coordinates. The spatial relationship between stalling events was examined by computing the distance between the first (most 5′) occurrence of Val and Ile stalling events within the same gene.

## Supplementary Information

Additional file 1. Fig. S1-S12: Supplementary Figures.

Additional file 2. Table S1: Dwell time changes upon BCAA starvation.

Additional file 3. Table S2: Free intracellular amino acid levels upon BCAA starvation.

Additional file 4. Table S3: GO terms and differential expression analysis of RNA-seq, Ribo-seq, RD and proteomic changes upon BCAA starvation.

Additional file 5. Table S4: Transcript specific polarity scores (distribution of ribosome density from 5′ to 3′).

Additional file 6. Table S5: Extracted positions and transcripts displaying ribosome stalling (identified by peak calling).

Additional file 7. Table S6: Reagents and oligo sequences.

## Data Availability

Raw sequencing data can be accessed through the GEO repository, accession numbers GSE291652 [92] and GSE291653 [93]. The mass spectrometry proteomics data have been deposited to the ProteomeXchange Consortium via the PRIDE [94] partner repository with the dataset identifier PXD067949 [95]. All analysis scripts and related data used in this study are publicly available on GitHub [96]: https://github.com/naef-lab/Worpenberg_2025_GB_BCAA_starvation/ under an open-source MIT license. A DOI-archived version of the scripts and related data is available on Zenodo: 10.5281/zenodo.17035796 [97]. Publicly available sequencing datasets used in this study: Ribosome profiling under Arginine and Leucine deprivation [98].
