# Supplementary material for: Codon-specific ribosome stalling reshapes translational dynamics during branched-chain amino acid starvation
Source: Genome Biol. 2025 Sep 27;26:315. doi: 10.1186/s13059-025-03800-6 (PMC12476630; doi:10.1186/s13059-025-03800-6)
Supplement: Supplementary file 1 — Additional file 1. Fig. S1-S12: Supplementary Figures. [file 13059_2025_3800_MOESM1_ESM.docx]

**Supplementary Figures**


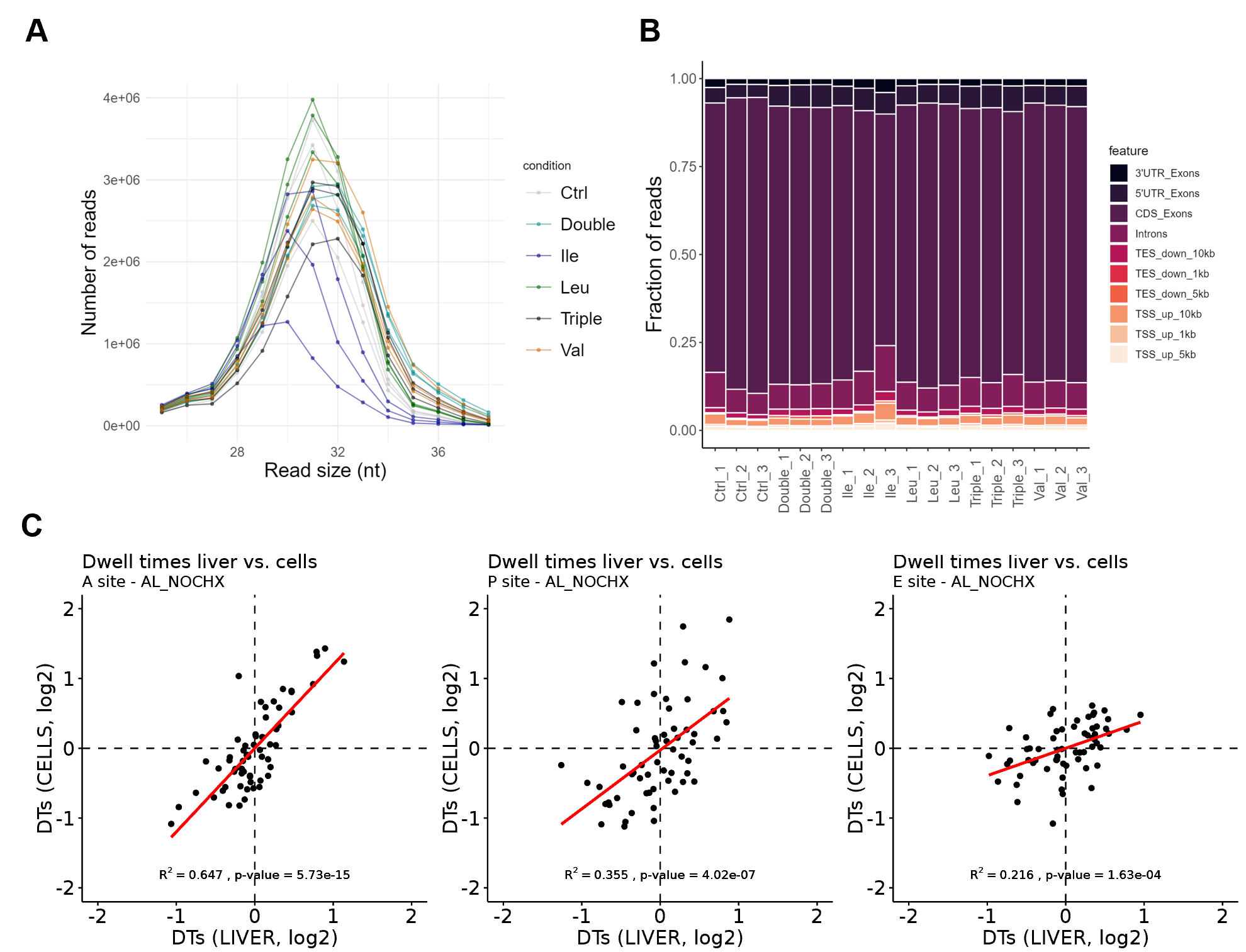


**Fig S1. Quality control and comparison of ribosome profiling data across experimental conditions.** (A) Footprint length distribution for each sample. (B) Percentage of mapped reads to different transcript features such as CDS and UTRs. (C) Scatter plots comparing dwell times (log₂) in liver [^1^](https://www.zotero.org/google-docs/?gocDkh) vs. cells (this study) at the ribosomal A, P, and E sites. Linear regression (red line) with R² and p-value displayed. Dashed lines indicate zero reference points.


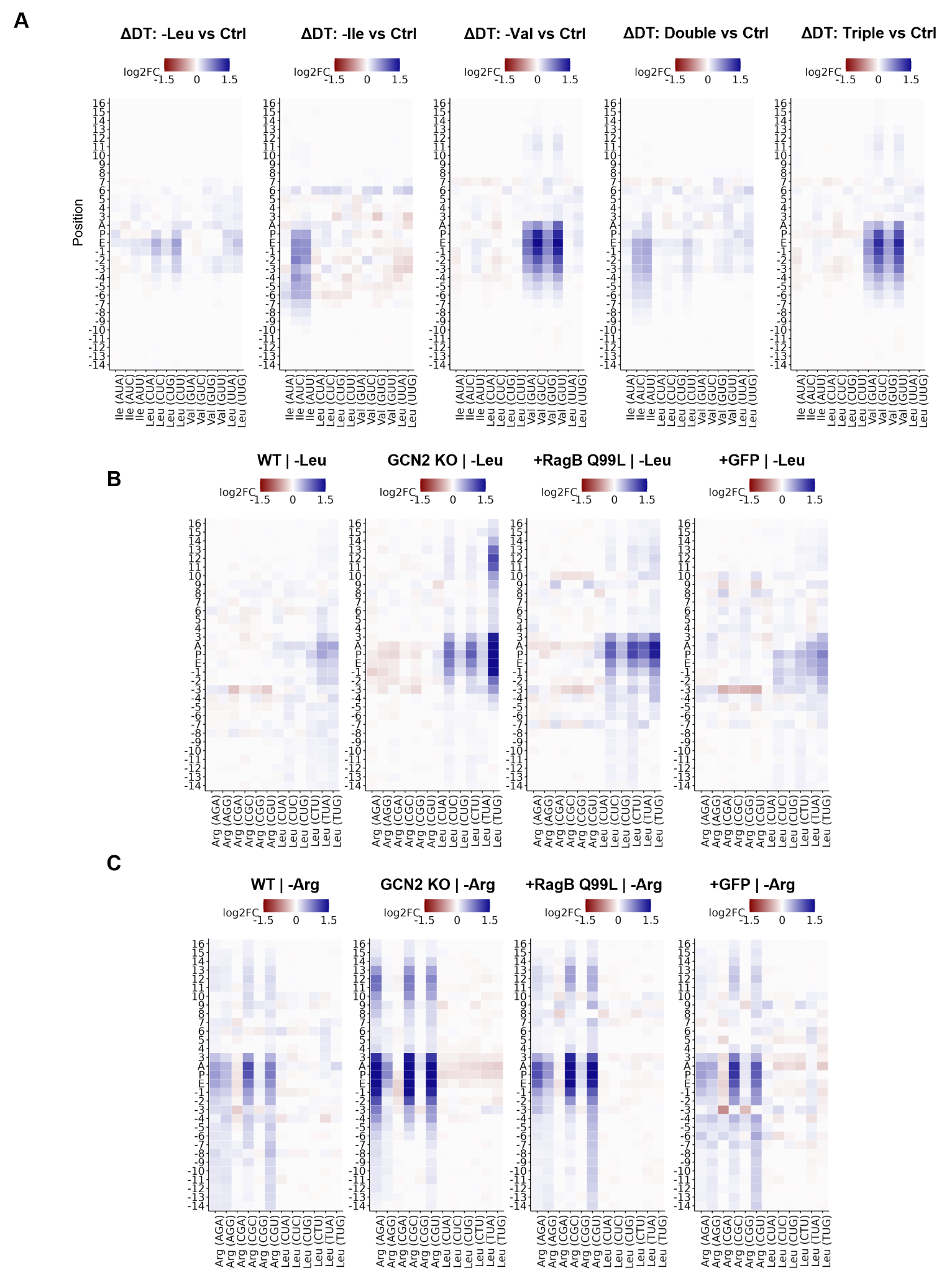


**Fig S2. Amino acid starvation alters ribosomal dwell times beyond the A site in mammalian cells.** (A-B) Heatmaps of ΔDT (log₂FC) relative to Ctrl for annotated codons across ribosomal positions (-14 to 18, including E, P, and A sites) across (A) BCAA starvations in NIH3T3 cells of this study, (B-C) DTs resulting from reanalysis of published Ribo-seq data [^2^](https://www.zotero.org/google-docs/?Dac4kQ), produced in HEK293T cells (Wildtype, GCNO KO, RagB mutant, GFP overexpression) starved for 6h of (B) leucine or (C) arginine.


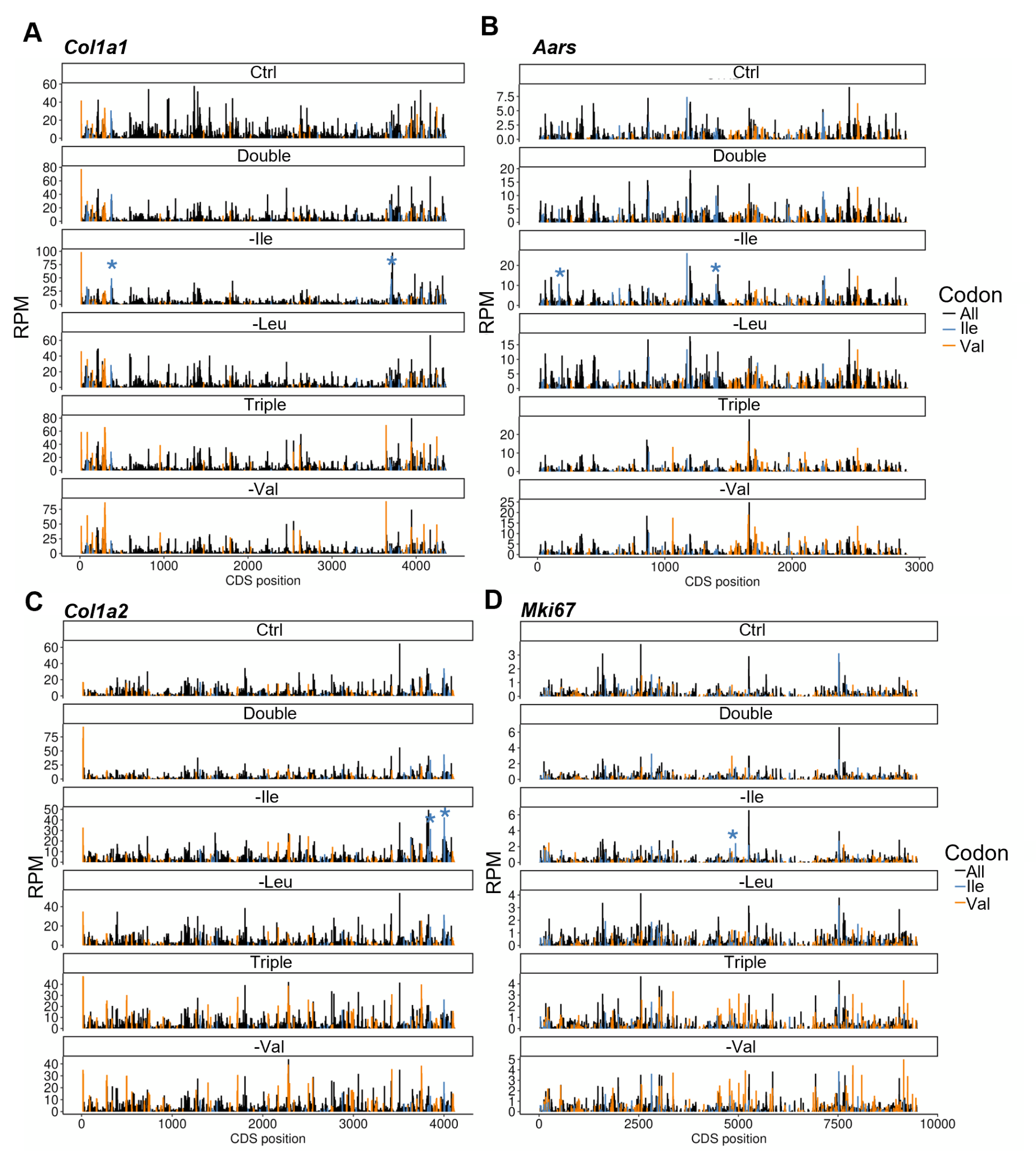


**Fig S3. Ribosome density profiles at the gene-level illustrate differential stalling across conditions. (A-D)** Representative ribosome profiling tracks for the transcripts of (A) *Col1a1*, (B) *Aars*, (C) *Col1a2* and (D) *Mki67* with Val and Ile codon positions (P-site ± 1) annotated. Exemplary positions with an Ile codon harbouring increased RPMs upon Ile starvation are marked with blue stars.

**
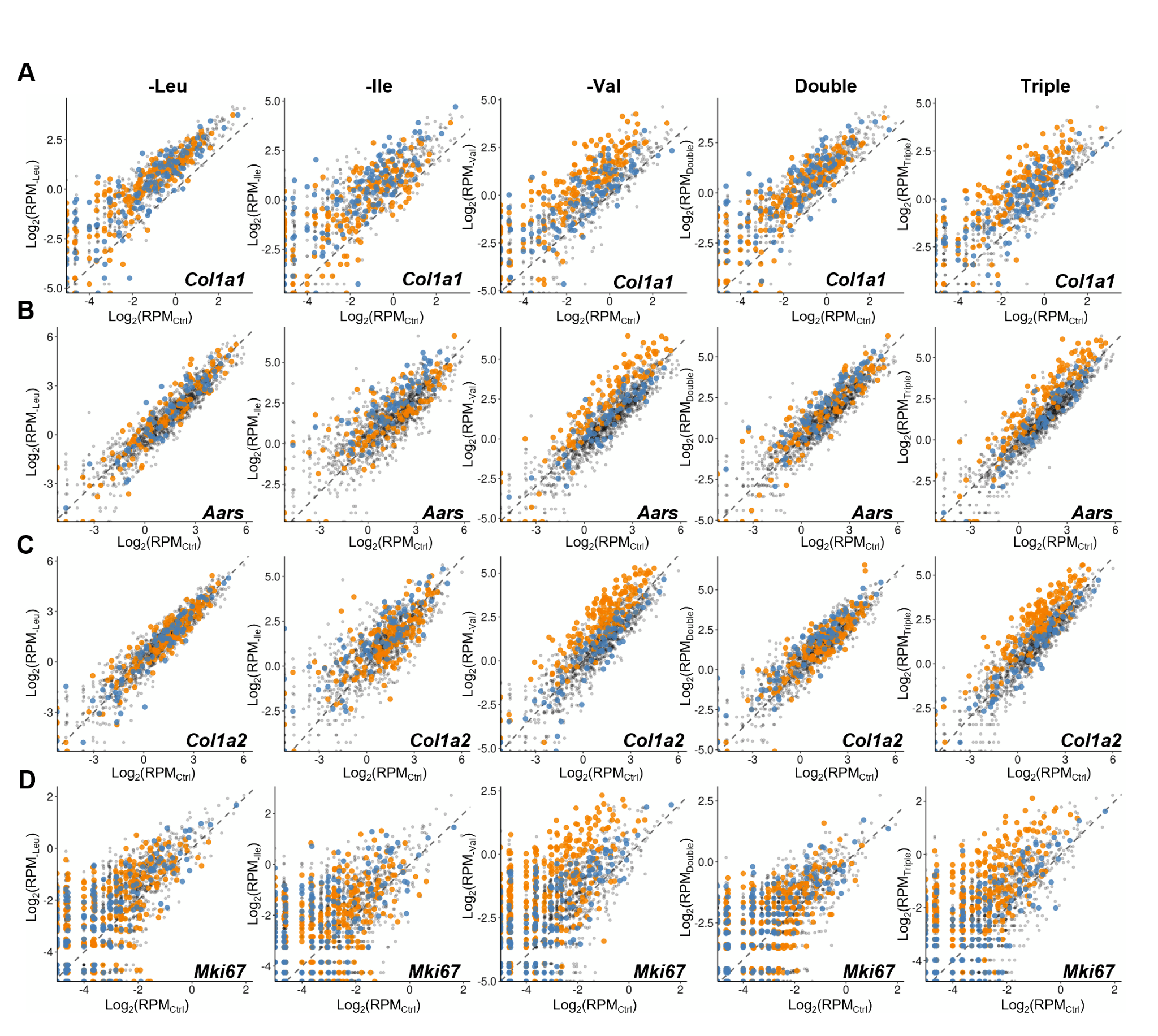
**

**Fig S4. Scatter plots illustrate differential stalling across conditions. (A-D)** Scatter plots of RPM count for every position in the control vs. indicated starvation condition for the transcripts of (A) *Col1a1,* (B) *Aars*, (C) *Col1a2* and (D) *Mki67* with Val (orange) and Ile (blue) codons marked.

**
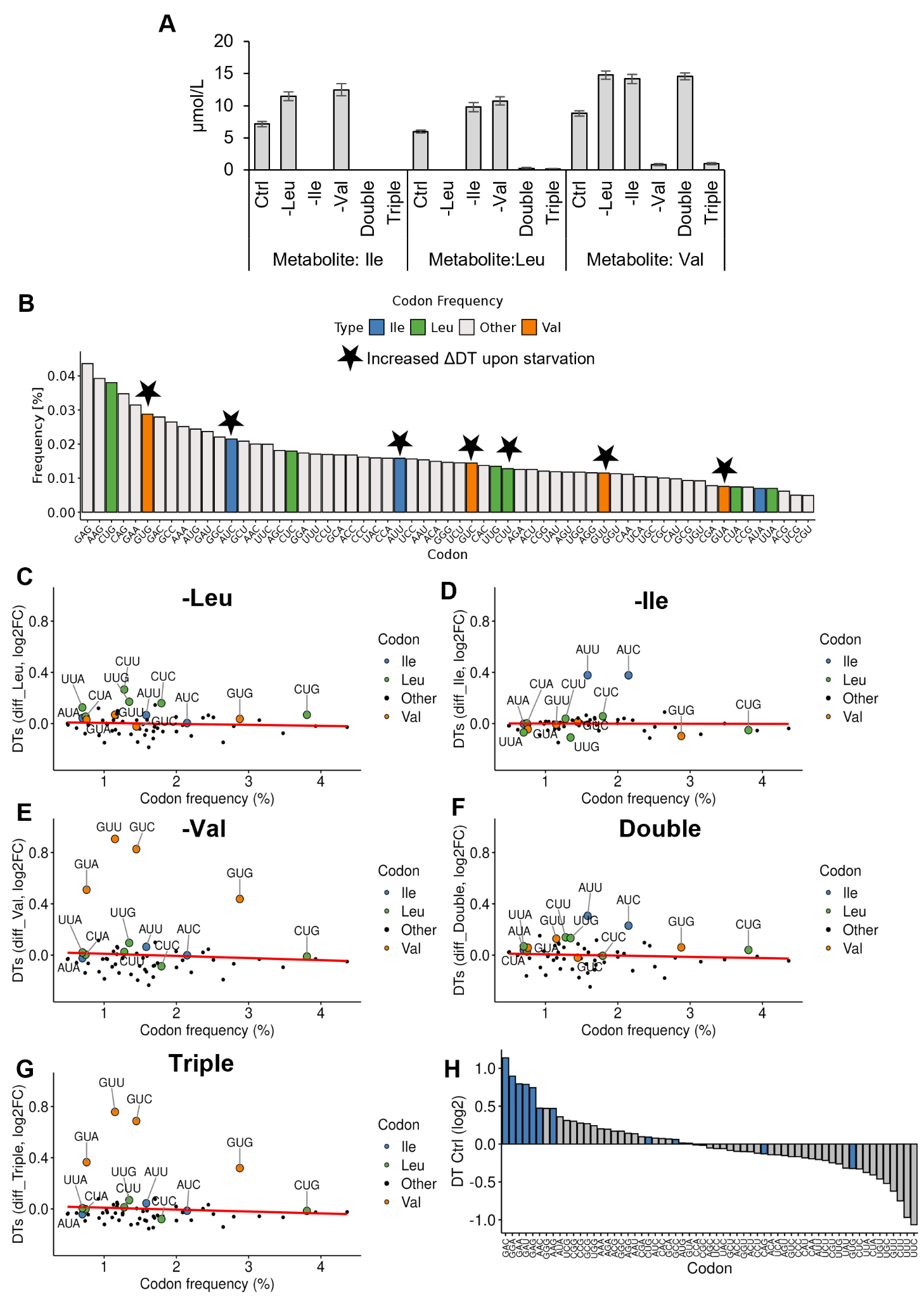
**

**Fig S5. Intracellular BCAA levels confirm effective starvation, and codon usage analyses reveal no correlation with stalling behavior. (A)** Quantification of intracellular free amino acid concentrations (Val, Ile, Leu) under the indicated starvation conditions. Bars represent the mean concentration in µmol/L across five biological replicates; error bars indicate inter-replicate variance. Missing bars indicate that measurements were below the detection threshold. **(B)** Bar plot showing codon frequencies in protein-coding transcripts expressed in NIH3T3 mouse fibroblasts. Codons for leucine (green), isoleucine (blue), and valine (orange) are highlighted. Frequencies are averaged across transcripts and sorted by descending frequency. Codons that exhibit significantly increased dwell times (DTs) under any starvation condition are marked with a star. **(C–G)** Scatter plots of codon frequency (%) versus change in ribosome dwell time (ΔDT) for each starvation condition. Codons for valine (orange), isoleucine (blue), and leucine (green) are highlighted. Red trendlines indicate that there is no significant correlation between codon frequency and ΔDT. **(H)** Bar plot showing the mean ribosome dwell time (DT) for each sense codon under control conditions, ranked from highest to lowest. Codons highlighted in blue were found at extracted stalling sites in cases where no hungry codon was present (see section “*Non-uniformly distributed codons create elongation bottlenecks”*).


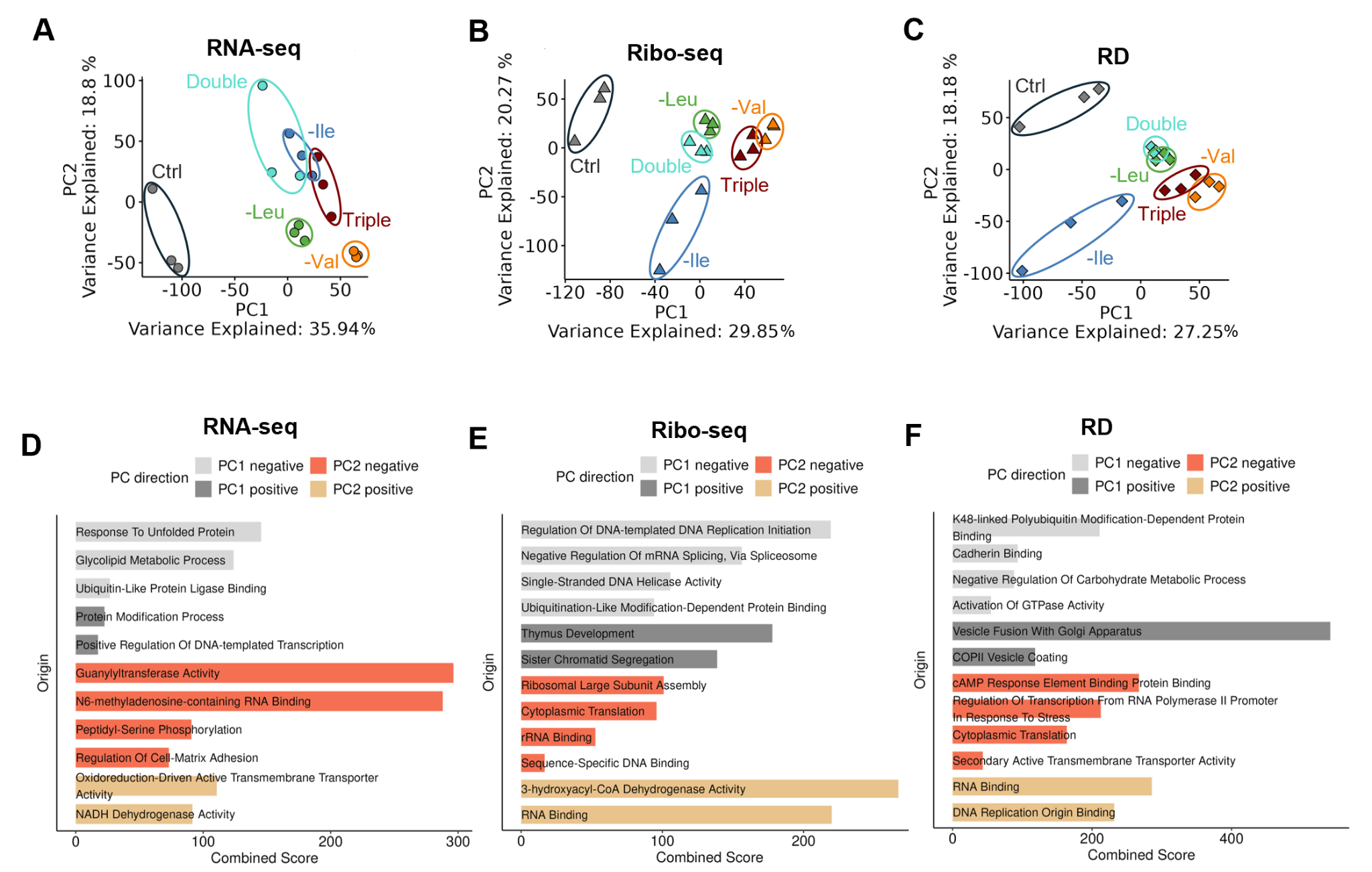


**Fig S6. Principal component analysis reveals distinct transcriptional and translational regulation across starvation conditions.** (A-C) Principal Component Analysis (PCA) of (A) RNA-seq, (B) Ribo-seq, and (C) ribosome densities (RD) data after group-wise mean-centering relative to Ctrl. PC1 vs. PC2 plot shows sample clustering based on gene expression variation. Colors represent treatment conditions, while shapes indicate data type. Variance explained by each principal component is displayed on the axes. (D-F) Top enriched GO terms for PC1 and PC2 (positive and negative loadings) from PCA analysis of (D) RNA-seq, (E) Ribo-seq and (F) RD. Transcripts included in the enrichment analysis were selected based on loading values exceeding ±15. GO terms were filtered for adjusted p-value < 0.05 and combined enrichment score > 15.


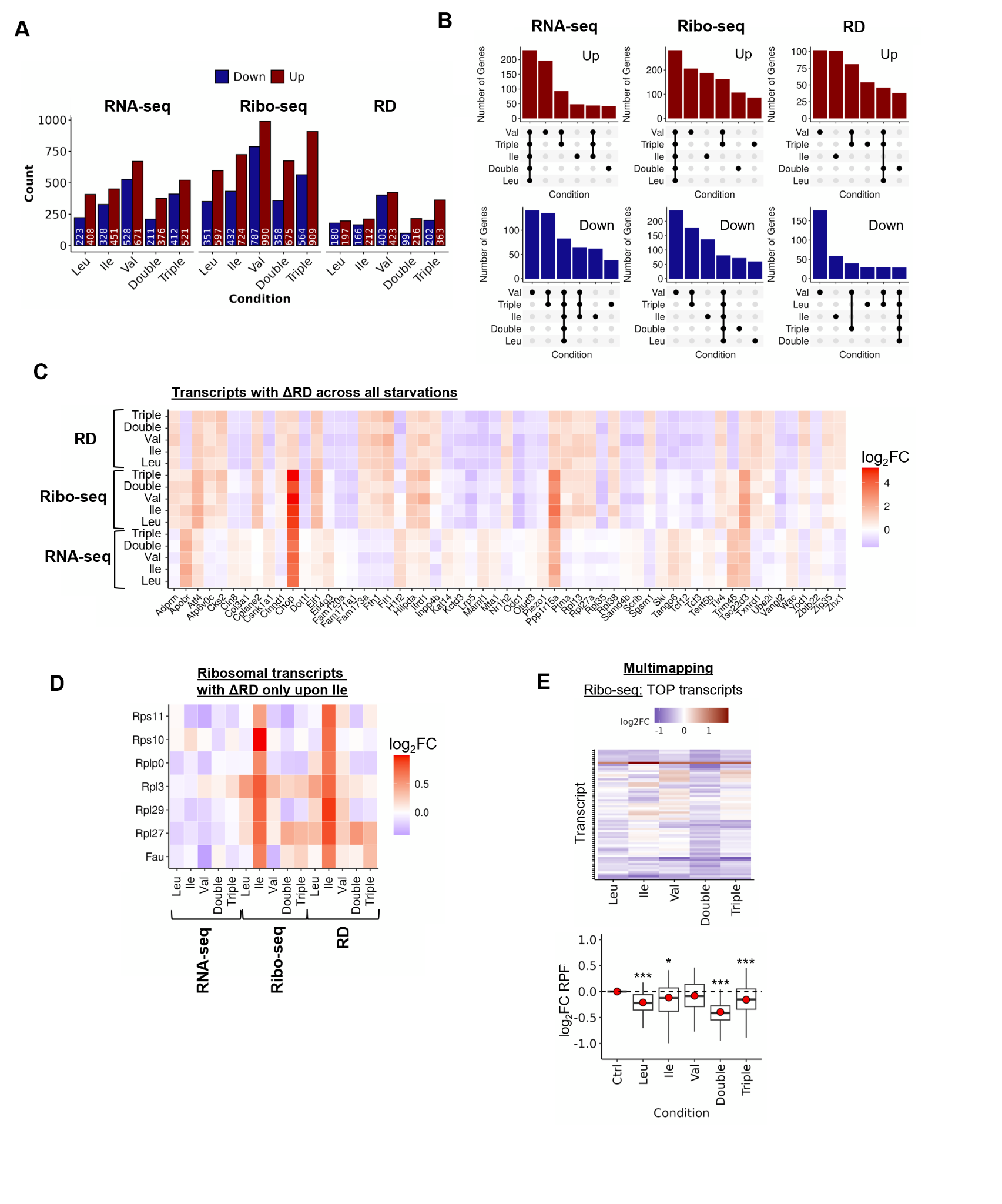


**Fig S7. Analysis of transcript-specific ribosome densities (RD) shows condition-specific patterns.** (A) Bar plot showing the number of significantly upregulated (red) and downregulated (blue) transcripts across different deprivation conditions (-Leu, -Ile, -Val, Double, Triple). Transcripts were classified as differentially expressed based on |log₂FC| > 0.58 with p < 0.05. Separate counts are shown for RNA-seq, Ribo-seq, and RD data. (B) UpSet plots illustrating the overlap of differentially expressed genes across deprivation conditions for RNA-seq, Ribo-seq, and RD datasets. Genes were considered significantly upregulated, downregulated based on |log₂FC| > 0.58 with p < 0.05. Bars indicate the number of genes shared across multiple conditions. (C) Heatmap displaying log_2_FC of transcripts with dysregulated ribosome density (RD) in all starvation conditions (upregulated or downregulated). (D) Heatmap displaying log_2_FC of a subset of transcripts that only show a significant RD change upon Ile starvation. Displayed are the transcript related to ribosomal proteins. (E) Heatmap and quantification of log₂FC for the known TOP motif containing transcripts[^3^](https://www.zotero.org/google-docs/?6KT3XI) in our Ribo-seq using unique and multimapping reads (see Material and Methods). Genes are clustered based on their expression profiles using k-means clustering (k = 12). In the boxplot, red points indicating the mean expression change. Significance was assessed using unpaired t-tests.


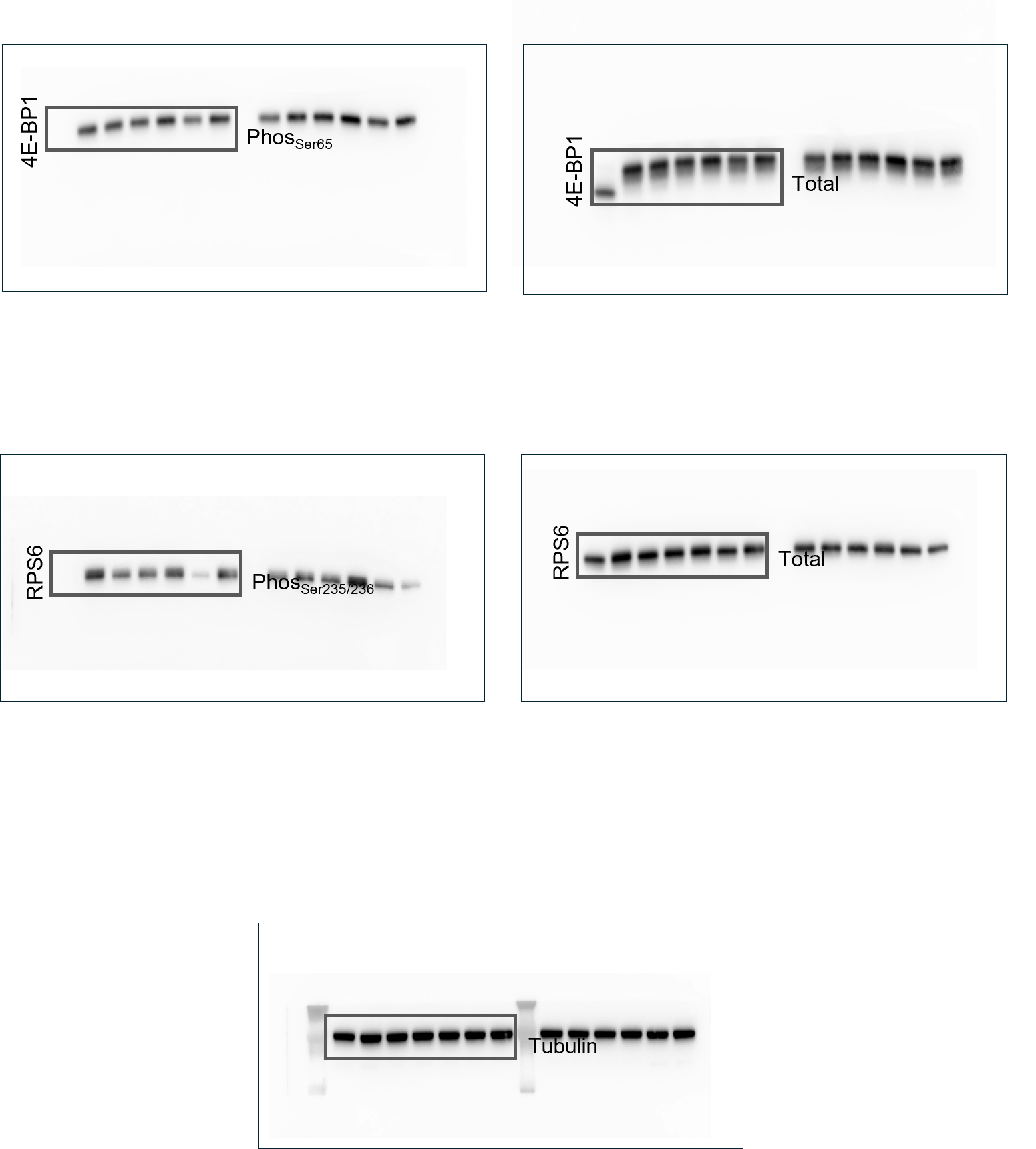


**Fig S8. Uncropped Western blots corresponding to Figure 2.**

Full scans of the Western blots shown in Figure 2, presented without cropping. Relevant lanes and bands used in the main figure are indicated.


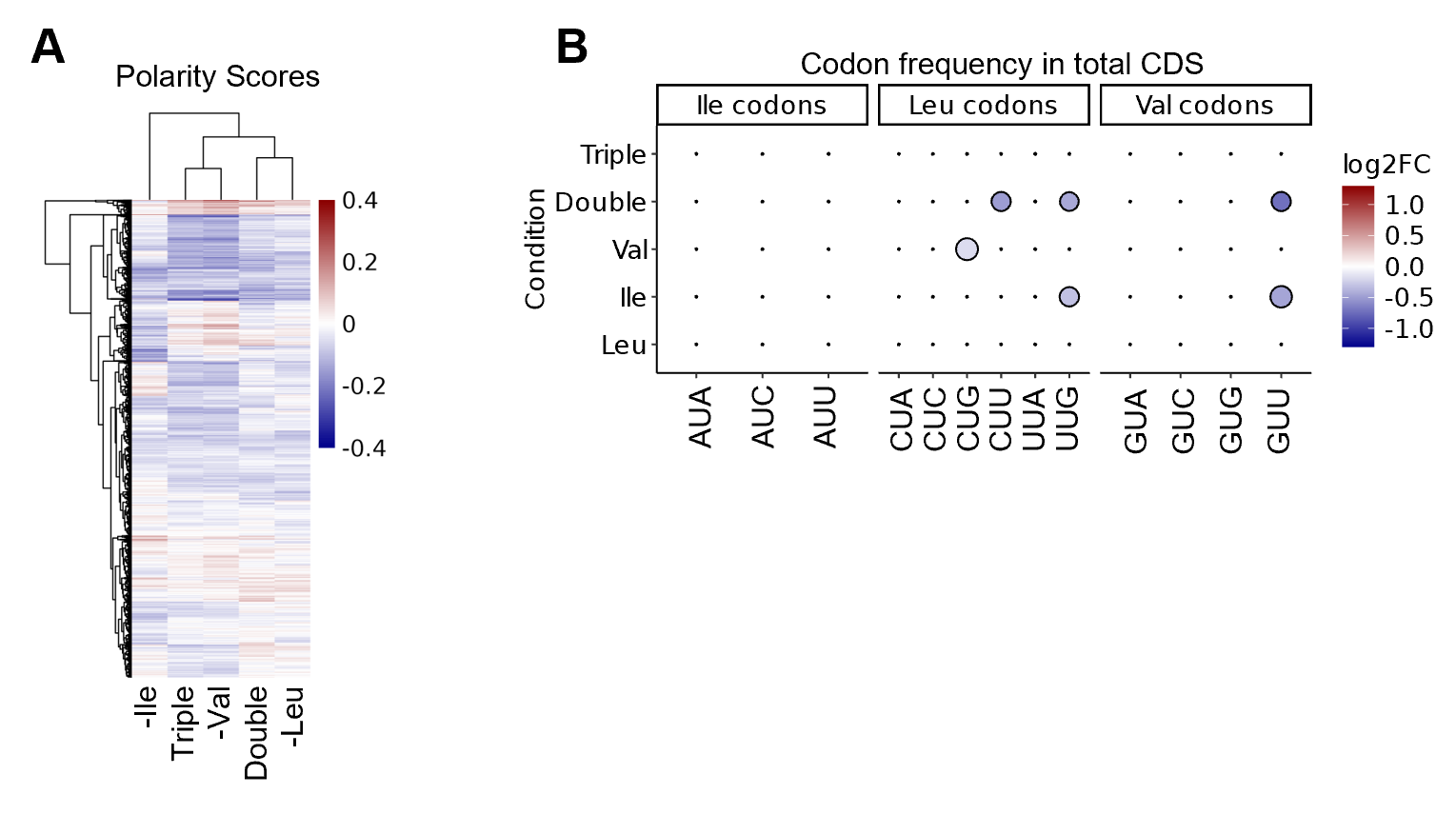


**Fig S9. Transcripts with polarity score shifts do not have an increased frequency of starved amino acid in total CDS.** (A) Heatmap of transcript-specific polarity score changes across the tested starvations relative to Ctrl. (B) Dot plot summarizing changes in normalized codon frequencies in groups of transcripts extracted in Figure 4D in their total CDS. For each codon group (Val, Leu, and Ile codons), the log_2_FC relative to all transcripts is plotted. Dot size reflects the -log₁₀(p-value) from unpaired t-tests (with BH adjustment), and the fill color indicates the magnitude and direction of change.


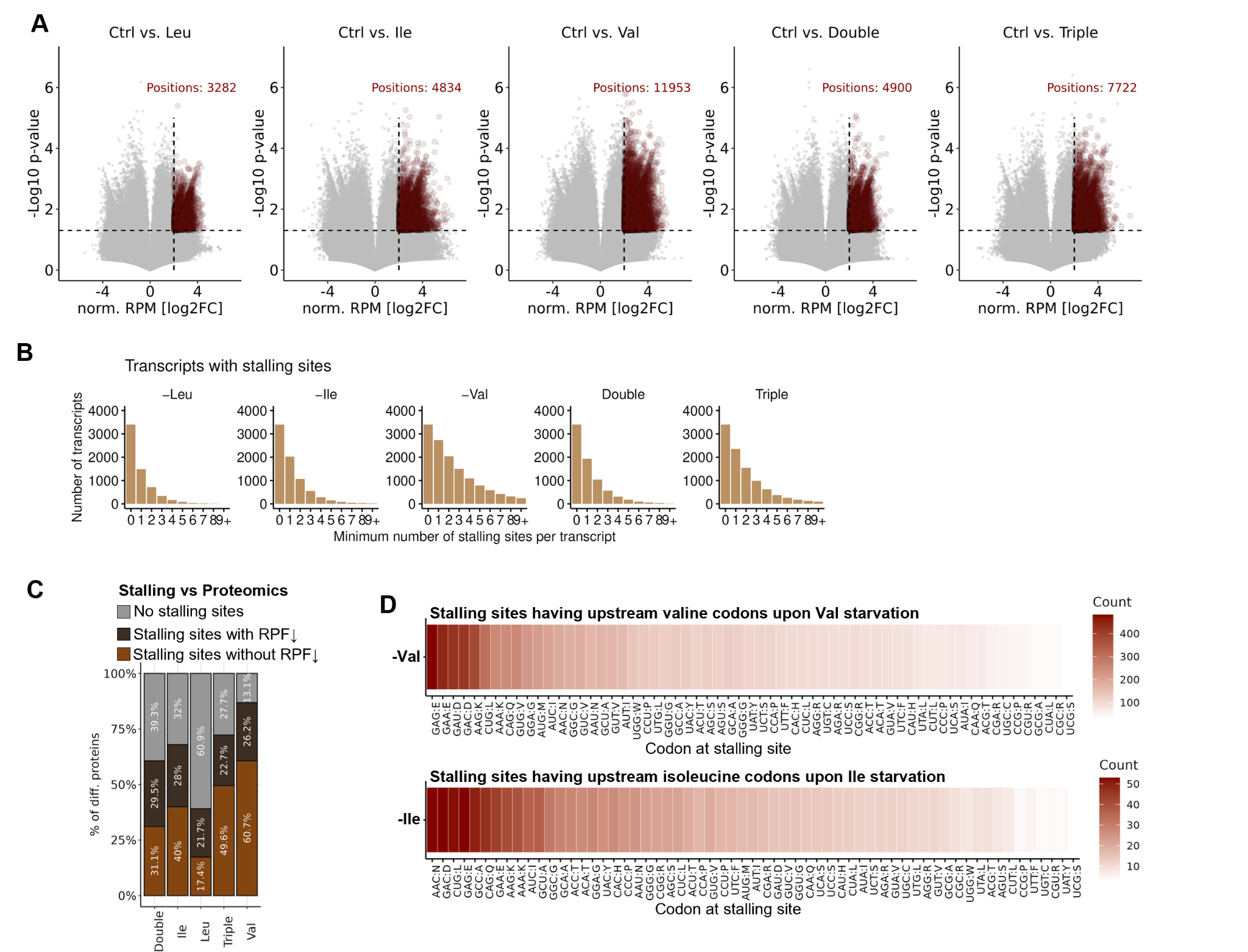


**Fig S10. Non-uniform codon distribution creates elongation bottlenecks.** (A) Volcano plots displaying log₂FC of RPM values measured by Ribo-seq versus -log₁₀(p-value) for each position within transcripts. The used RPM values are normalized to the total counts on each transcript. Positions meeting peak-calling criteria for significant upregulation are highlighted in red. (B) Number of transcripts with at least the indicated number of ribosome stalling sites under each condition. The first bar (0) represents the total number of transcripts, irrespective of stalling. (C) Comparison of downregulated proteins in each starvation to transcripts harbouring stalling sites (as identified in 5A). Gray bar indicates the percentage of downregulated proteins without stalling site and brown indicates proteins with an identified stalling site, with dark brown indicating an additionally downregulated RPF level (measured by Ribo-seq) and bright brown indicating no corresponding downregulated RPF level. (D) Heatmap illustrates the frequency of specific codons at stalling sites, conditioned on the presence of valine (Val) or isoleucine (Ile) codons in the 1–3 positions upstream of the stalling site.

**
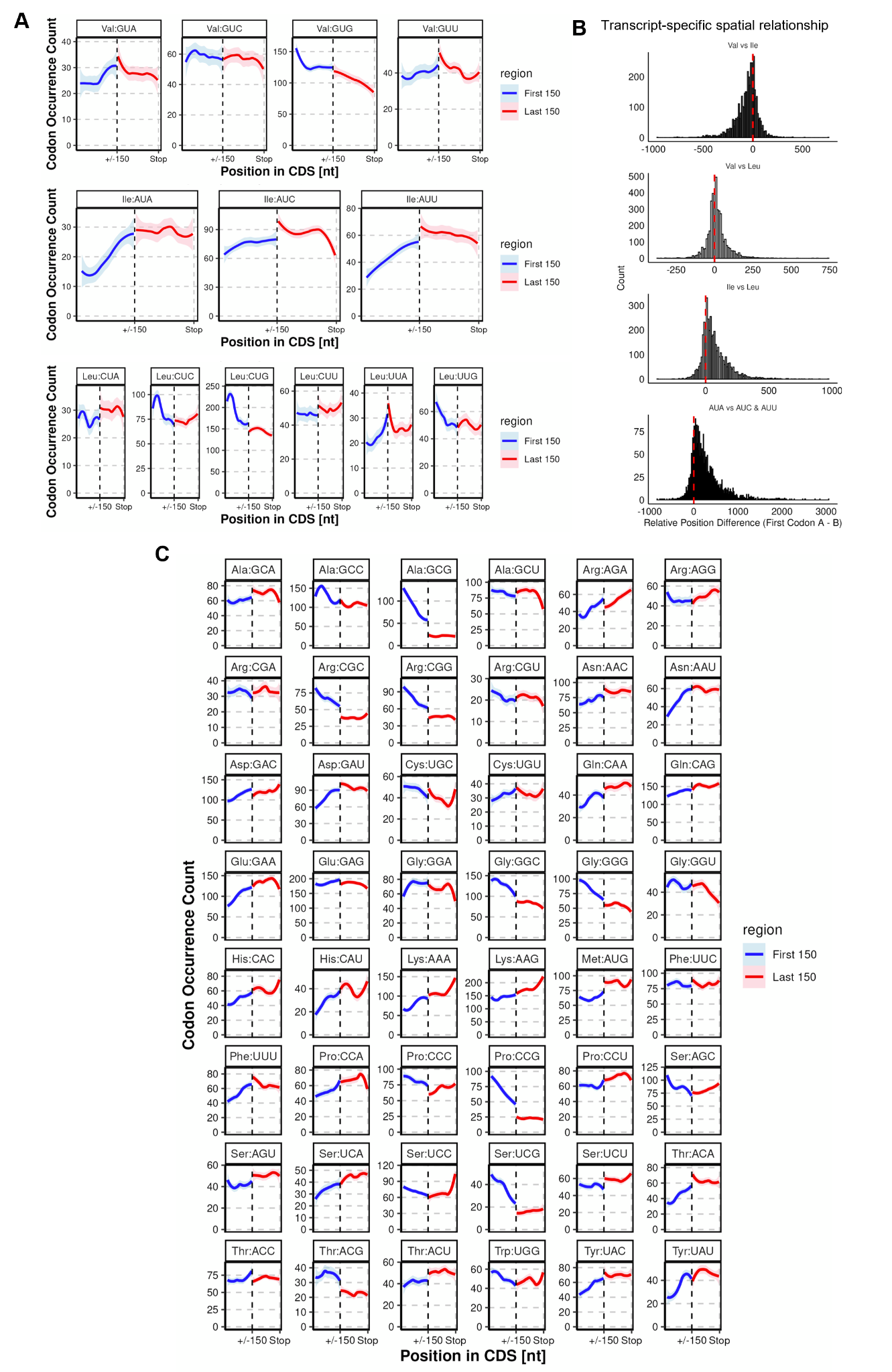
**

**Fig S11. Codon frequencies at the 5′ and 3′ ends of coding sequences.** (A) Frequencies of valine (Val), isoleucine (Ile) and leucine (Leu) codons in the first and last 150 codons across all transcripts. (B) Shown are pairwise comparisons of the codon positions within coding sequences (CDSs), specifically the distance between the first Val codon and the first Ile codon, the first Val and the first Leu codon, and the first Ile and first Leu codon. In addition, the position of the first AUA codon (Ile) is compared to the combined first occurrence of AUC and AUU codons (Ile). (C) Plots showing the codon count of each sense codon (excluding Val, Ile, and Leu codons) in the first (blue) and last (red) 150 codons across all expressed transcripts. This analysis highlights positional biases in codon usage within coding sequences.


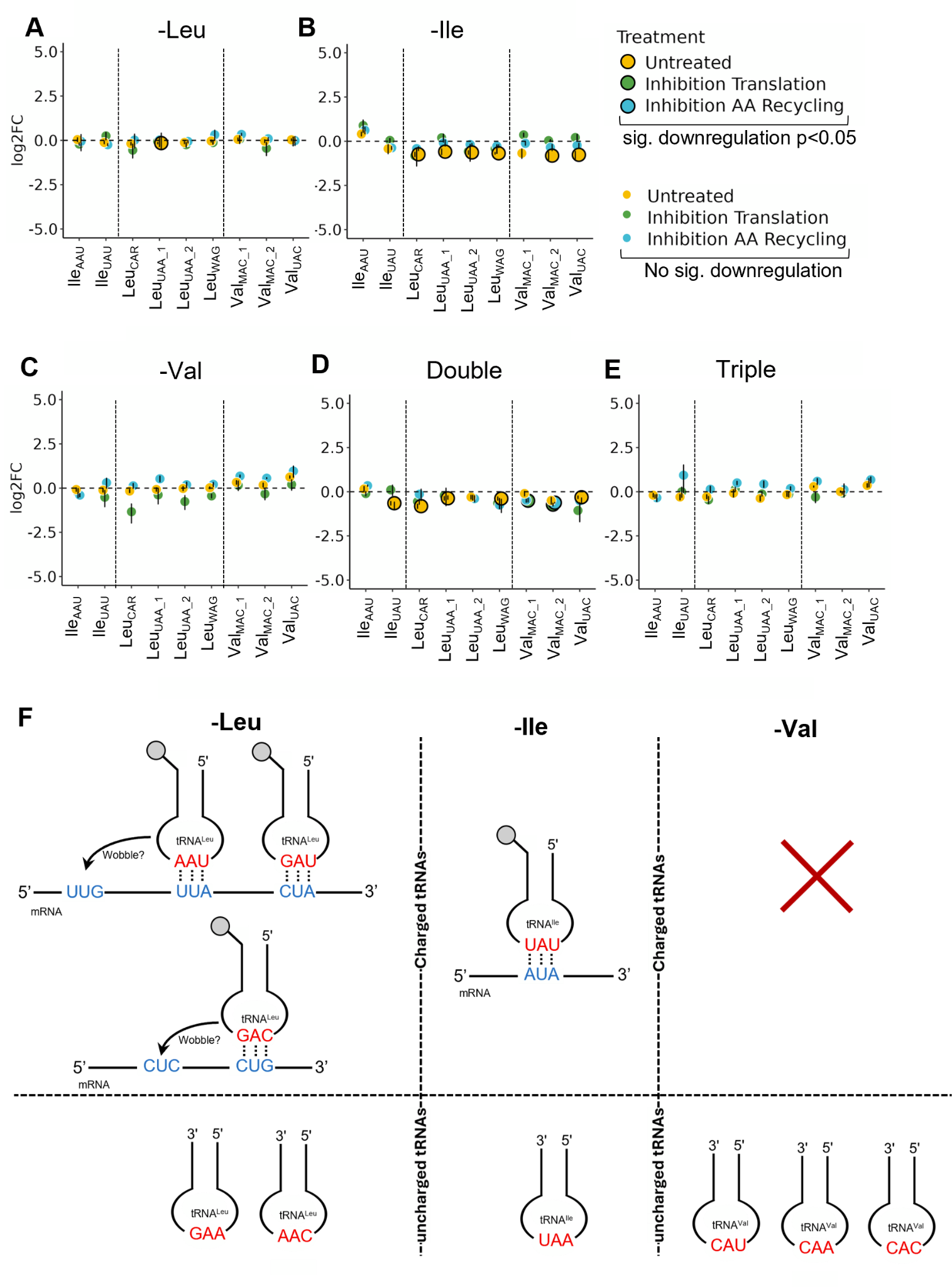


**Fig S12. Total tRNA levels show minimal changes across starvation conditions.** (A-E) Differential tRNA isoacceptor level under the indicated starvation conditions. Yellow indicates starved cells without treatment. Green represents cells treated with 100 µg/mL cycloheximide (CHX) in the last 30 min of starvation to inhibit translation. Blue represents cells treated with 10 µM MG132 and 160 nM Bafilomycin A1 to inhibit proteasomal and autophagic degradation (amino acid recycling inhibition). Certain tRNA isodecoders could not be measured separately and are therefore represented with IUPAC ambiguity codes: W (A or U), R (A or G), and M (A or C). Large outlined dots indicate significant downregulation. Sample sizes: untreated (n = 5), translation and AA recycling inhibition (n = 2). (F) Schematic overview of tRNA isoacceptor charging patterns observed under individual (-Leu, -Ile, -Val) starvation. Codons that remain efficiently decoded under starvation are shown in blue and linked to their charged decoding tRNAs. Arrows indicate wobble base pairing where applicable.

**Supplementary References**

[1. Gobet, C. *et al.* Robust landscapes of ribosome dwell times and aminoacyl-tRNAs in response to nutrient stress in liver. *Proc. Natl. Acad. Sci. U. S. A.* **117**, 9630–9641 (2020).](https://www.zotero.org/google-docs/?AdfsBR)

[2. Darnell, A. M., Subramaniam, A. R. & O’Shea, E. K. Translational Control through Differential Ribosome Pausing during Amino Acid Limitation in Mammalian Cells. *Mol. Cell* **71**, 229-243.e11 (2018).](https://www.zotero.org/google-docs/?AdfsBR)

[3. Thoreen, C. C. *et al.* A unifying model for mTORC1-mediated regulation of mRNA translation. *Nature* **485**, 109–113 (2012).](https://www.zotero.org/google-docs/?AdfsBR)
